# RNA Sequencing of Lens Capsular Epithelium Implicates Novel Pathways in Pseudoexfoliation Syndrome

**DOI:** 10.1167/iovs.63.3.26

**Published:** 2022-03-29

**Authors:** Sean Mullany, Henry Marshall, Tiger Zhou, Daniel Thomson, Joshua M. Schmidt, Ayub Qassim, Lachlan S. W. Knight, Georgina Hollitt, Ella C. Berry, Thi Nguyen, Minh-Son To, David Dimasi, Abraham Kuot, Joshua Dubowsky, Rhys Fogarty, Michelle Sun, Luke Chehade, Shilpa Kuruvilla, Devaraj Supramaniam, James Breen, Shiwani Sharma, John Landers, Stewart Lake, Richard A. Mills, Mark M. Hassall, Weng O. Chan, Sonja Klebe, Emmanuelle Souzeau, Owen M. Siggs, Jamie E. Craig

**Affiliations:** 1 Flinders Centre for Ophthalmology, Eye and Vision Research, Flinders Health and Medical Research Institute (FHMRI), Flinders University, Adelaide, Australia; 2 SAHMRI Bioinformatics Core, South Australian Health and Medical Research Institute, Adelaide, Australia; 3 Flinders Department of Pathology, Flinders Health and Medical Research Institute (FHMRI), Flinders University, Adelaide, Australia; 4 Garvan Institute of Medical Research Institute, Darlinghurst, Sydney, Australia

**Keywords:** pseudoexfoliation, transcriptomics, RNAseq

## Abstract

**Purpose:**

Pseudoexfoliation syndrome (PEX) is a common systemic disease that results in severe and often irreversible vision loss. Despite considerable research effort, PEX remains incompletely understood. This study sought to perform the first RNAseq study in elucidate the pathophysiology of PEX, and contribute a publicly available transcriptomic data resource for future research.

**Methods:**

Human ocular lens capsular epithelium samples were collected from 25 patients with PEX and 39 non-PEX controls undergoing cataract surgery. RNA extracted from these specimens was subjected to polyadenylated (mRNA) selection and deep bulk RNA sequencing. Differential expression analysis investigated protein-coding gene transcripts. Exploratory analyses used pathway analysis tools, and curated class- and disease-specific gene sets.

**Results:**

Differential expression analysis demonstrated that 2882 genes were differentially expressed according to PEX status. Genes associated with viral gene expression pathways were among the most upregulated, alongside genes encoding ribosomal and mitochondrial respiratory transport chain proteins. Cell adhesion protein transcripts including type 4 collagen subunits were downregulated.

**Conclusions:**

This comparative transcriptomic dataset highlights novel and previously recognized pathogenic pathways in PEX and provides the first comprehensive transcriptomic resource, adding an additional layer to build further understanding of PEX pathophysiology.

Pseudoexfoliation syndrome (PEX) is a common systemic disease characterized by the extracellular accumulation of highly insoluble fibrillar material.^1^^,^^2^ Deposition of these fibrillar structures can be visualized in the anterior chamber of the eye at the iris pupil margin, on the surface of the anterior lens capsule, and within the iridocorneal angle. Epidemiological studies have demonstrated PEX predominantly affects individuals aged 40 years or more, with reported prevalence rates of 2% to 22% in Europe.^3^ Although PEX is associated with various pathological systemic features, the most clinically relevant manifestations are the ocular features, which include cataract and secondary open-angle glaucoma.^2^ An increased prevalence of age-related cataract is an important disease consequence,^4^^–^^6^ with Australian epidemiological data demonstrating that PEX-affected eyes have twice the prevalence of nuclear cataract.^6^ Secondary open-angle glaucoma, which occurs in up to 44% of PEX-affected eyes,^7^ is typically severe, with progressively increasing IOP, rapid vision loss, and poor response to medical therapy.^8^ Cataract surgery in PEX is associated with higher intraoperative complication rates owing to zonular instability, lens dislocation, impaired pupillary dilation, and increased postoperative complication rates owing to corneal decompensation, IOP spikes, and posterior capsular opacification.^9^

As a disease phenomenon, PEX is generally regarded as an age-related, fibrotic process.^2^ The current body of literature suggests that cellular and oxidative stress result in altered TGFB1 signaling, microfibrillopathy, and precipitation of elastic microfibrils.^2^ Elucidating the pathobiological processes underpinning PEX has been a major area of recent research interest. Genome-wide association studies (GWAS) have identified strong associations between PEX and common genetic variants in several genes, most notably lysyl oxidase like 1 (*LOXL1*), which codes for an enzyme involved in the cross-linking of collagen and elastin.^10^^–^^19^ More recently, an exome-wide association study identified disease associations with rare coding variants in the cytochrome P450 gene *CYP39A1*.^20^ Additional attempts to understand PEX include targeted protein/proteomic analyses, which have identified more than 70 constituents of PEX material, and targeted gene expression studies, which have implicated multiple disordered biological processes in PEX.^2^^,^^21^^–^^35^ Despite advances in our understanding of the genetic, structural, and functional architecture of this disease, the mechanisms by which PEX results in its various ocular disease manifestations remain unclear. Given these limitations to our current understanding, there is a need for further experimental data, especially from novel omics-based analyses.^36^

In this study, a comparative RNA sequencing (RNAseq) analysis of primary lens capsule epithelium from PEX and non-PEX cases was undertaken to elucidate the transcriptional architecture of this disease. The data was also utilized to assess the expression of genes previously implicated in the ocular sequelae of PEX.

## Methods

### Experimental Design

A case-control study design was used to investigate the transcriptional architecture of PEX through bulk RNASeq analysis of mRNA in surgically excised lens epithelium, one of the cell types affected by PEX, which is also believed to produce fibrillar PEX material).^1^^,^^37^^,^^38^ The lens capsular epithelium consists of a single epithelial cell layer bounded anteriorly by the lens capsule—an acellular basement membrane—and posteriorly by the crystalline lens. Lens capsular epithelium samples were collected from age- and gender-matched PEX and non-PEX patients undergoing routine cataract surgery for visually significant cataracts with a predominant nuclear sclerotic phenotype. Given the paucity of published transcriptional data, a primarily agnostic approach was undertaken to elucidate disease pathways using common pathway analysis tools. To identify transcriptional changes within gene classes identified in the current study, and from previously published data, further analyses were performed investigating curated and manually annotated class-based gene sets. To identify possible associations between PEX and its common pathological ocular sequelae, similar analyses were performed with gene lists comprising Mendelian and GWAS disease-associated genes associated with cataract, PEX, glaucoma, and zonular instability.

### Ethical Approval

Ethics approval was obtained through the Southern Adelaide Clinical Human Research Ethics Committee (HREC 186.09). This study adhered to the tenets of the revised version of the Declaration of Helsinki, and followed the National Health and Medical Research Council statement of ethical conduct in research involving humans.

### Subjects

Participants included patients, with or without PEX, undergoing cataract surgery for visually significant cataract. PEX was defined as the accumulation of characteristic whitish material deposits on the anterior lens capsule and at the pupillary margin. A diagnosis of PEX was made by an ophthalmologist on dilated slit lamp examination. A PEX cohort was age and gender matched with non-PEX controls, which were selected for RNAseq from a larger collection. A PEX cohort was age and gender matched with non-PEX controls who were selected for RNAseq from a larger collection. To address potential biases associated with both disease asymmetry, we sampled only one eye from each participant. Non-PEX eyes from individuals with a history of contralateral PEX were excluded. To minimize confounding, less common phenotypes, including cortical spoke and posterior subcapsular cataracts, were excluded from both cohorts.

Because PEX is commonly associated with both elevated IOP and glaucoma, further classification was performed based upon the presence of any previously recorded IOP of 25 mm Hg or higher (using Goldmann applanation tonometry) in the study eye, with a corresponding glaucomatous visual field defect. These participants were subclassified as high-tension glaucoma (HTG), irrespective of their PEX status. Participants with a history of normal-tension glaucoma, inflammatory eye disease, AMD, diabetic retinopathy, or previous intraocular surgery in the study eye were excluded.

### Sample Collection

Anterior lens capsular epithelium samples were collected as capsulorhexis specimens from patients during routine cataract surgery. Samples were removed under sterile conditions, immediately placed in RNAlater solution (ThermoFisher Scientific, Waltham, MA) and stored at 4°C for 7 days before being archived at −80°C for later use.

### RNA Extraction and Purification

Lens capsule samples were thawed at room temperature before RNA extraction using an RNeasy Plus Mini kit (Qiagen, Hilden, Germany) according to the manufacturer's protocols. RNA concentration was measured using a Qubit 2.0 Fluorometer (ThermoFisher Scientific), and quality was assessed with an Agilent Tapestation (Santa Clara, CA) using the RNA ScreenTape chip. Samples with low RNA integrity numbers of less than 6.5 were excluded.

### Library Preparation and Sequencing

Total RNA was used to generate strand-specific Illumina-compatible sequencing libraries using the Tecan Universal Plus mRNA-Seq library kit (Tecan, Mannedorf, Switzerland) per the manufacturer's instructions (M01442 v2). Briefly, 125 ng of total RNA was polyA selected and fragmented before reverse transcription and second strand cDNA synthesis using dUTP. The resultant cDNA was end-repaired before the ligation of Illumina-compatible barcoded sequencing adapters. The cDNA libraries were strand selected and amplified for 12 polymerase chain reaction cycles (37°C for 10 minutes, 95°C for 2 minutes, 2× (95°C for 30 seconds, 60°C for 90 seconds), 12× (95°C for 30 seconds, 65°C for 90 seconds), 65°C for 5 minutes, held at 4°C) before assessment by Agilent Tapestation for quality, and Qubit fluorescence assay for quantity. Sequencing pools were generated by mixing equimolar amounts of compatible sample libraries based on Qubit measurements. Sequencing of the library pool was performed on an Illumina Novaseq 6000 S2 flow cell using paired-end 2 × 50 bp sequencing chemistry. All 64 raw FASTQ files were deposited to the Sequence Read Archive (SRA; Accession ID: PRJNA764775; http://www.ncbi.nlm.nih.gov/bioproject/764775).

### Bioinformatic Analysis Pipeline

High-throughput sequencing analysis was carried out through a South Australian Genomics Centre developed workflow initialized using the nextflow workflow language (https://github.com/sagc-bioinformatics/nf-rnaseq-sagc). Quality control was carried out using the FastQC tool, and raw reads were aligned to the GRCh38 reference genome using STAR v2.7. After alignment, mapped sequence reads were summarized to GRCh38 gene intervals using the *featurecounts* tool from the *RSubread* package.^39^ All reads mapping to a gene were considered, regardless of their presence in canonical or non-canonical transcripts. Multimapped reads were excluded using the default command *countMultiMappingReads = FALSE*. Samples with a total mapped read count of greater than 2 standard deviations (σ) from the mean mapped read counts per sample were excluded from further analyses.

### Differential Expression

All downstream analyses were performed in R (v4.0.3, RCore Team, Austria) using open-access packages. Differential expression analysis was performed using the *DESeq2* package on raw count tables filtered to exclude both non–protein-coding transcripts and X and Y sex chromosome transcripts.^40^ In brief, a differential expression object was generated by applying the *DESeqDataSetFromMatrix* command to a table containing raw transcript read counts per gene across samples. Primary PEX analysis was performed comparing PEX with non-PEX samples, and a secondary analysis was performed comparing HTG with no glaucoma samples. Independent filtering was performed using the default *results* function to remove outliers and genes with low read counts. A differential expression results table was generated by applying the *DESeq* command to these differential expression objects. Multiple testing correction was performed to generate adjusted *P* values for each differentially expressed gene (DEG) using the Benjamini-Hochberg method as part of the *DESeq2* package differential expression analysis. Differential expression in this study was defined by an absolute expression fold change of ±20% (i.e., a log_2_ fold change of ±log_2_1.2) in cases referenced against controls. Significance was defined by an adjusted *P* value threshold of less than 0.05. A principal components analysis (PCA) was performed using the *PCAtools* package. Differential gene expression plots were produced using the *ggplot2*, *EnhancedVolcano*, and *pheatmap* tools.

### Pathway Analysis

The pathways analysis involved a gene set enrichment of pathways from the Gene Ontology (GO) and Kyoto Encyclopaedia of Genes and Genomes (KEGG) datasets acquired from the Molecular Signatures Database (MSigDB v7.2.) using the *msigdbr* package. Gene set enrichment was performed using the *GSEA* function from the *clusterProfiler* package. Briefly, this function determines an enrichment score that quantifies how over-represented an annotated gene set is within a ranked gene list.^41^ Within this analysis, differential expression was defined by adjusted *P* value alone as further filtration using log_2_ fold thresholds would result in misclassification of genes that are significantly different between groups at magnitudes of less than log_2_1.2. A normalized enrichment score is calculated by adjusting the raw enrichment score for the size of each individual gene set.^42^ Multiple testing corrections were applied using the Benjamini–Hochberg method and an adjusted *P* value threshold was defined by a *P* value of less than 0.05. To account for large lists of enriched pathways, pruning was performed using the web-based *Revigo* tool, which summarizes lists of GO terms using a clustering algorithm which assesses for semantic similarity between identified terms. The Revigo tool does not fully account for pathway intersection as individual DEGs are not correlated between enriched pathways. The default correlation threshold (*C* = 0.7) was set when applying this tool (http://revigo.irb.hr/).^43^

### Gene Class Analysis

Class-specific gene sets accessed through the HGNC database (HUGO Genome Nomenclature Committee; https://www.genenames.org/) were used for targeted analysis of gene groups defined by functional subtypes. We selected individual gene sets to investigate gene classes implicated through our initial pathway analysis, and gene classes implicated in previous studies investigating the pathophysiology of PEX. In the absence of a HUGO gene set for the unfolded protein response, we used the hallmark unfolded protein response gene list from the REACTOME database (accessed from MSigDB v7.2 using *msigdbr*; see Appendix for gene lists and accession resources). To investigate expression perturbations within these gene sets, a semiquantitative enrichment analysis was performed. We used χ^2^ tests to investigate the number of genes within each gene class that were differentially expressed in the predominant direction of expression within that gene class relative to the full differential expression dataset. No multiple testing thresholds were applied to this analysis; therefore, nominal *P* values were reported.

### Disease Gene Analysis

We compiled disease gene lists from consensus resources to investigate the transcriptional profile of genes associated with PEX and its pathological ocular manifestations including cataract, glaucoma, and zonular instability. Differential expression of cataract disease-associated genes was performed using a list of Mendelian cataract genes compiling all known genes associated with isolated or syndromic Mendelian cataract acquired from CAT-MAP (Washington University School of Medicine, St Louis; https://cat-map.wustl.edu/).^44^ A pseudoexfoliation GWAS genes list included all genes with common variants identified by GWAS, and associated at genome-wide significance with PEX,^10^^–^^12^^,^^15^^–^^17^^,^^19^^,^^45^ with the notable addition of *CYP39A1*: rare variants of which were recently reported to be associated with PEX in an exome-wide rare variant association study.^20^ A glaucoma GWAS genes list included genes collocated with 127 glaucoma single nucleotide polymorphisms previously demonstrated to be associated with POAG risk across ancestries.^46^ Finally, we generated an ectopia lentis gene list from Mendelian diseases associated with ectopia lentis (a disease phenotype characterized by zonular instability) using data from the Online Mendelian Inheritance in Man (OMIM) database (see the Appendix for gene lists and accession resources). These analyses reported summary statistics from the primary differential gene expression analysis.

### Pseudoexfoliation Material Genes and Previous Expression Data Analysis

A PEX material gene list was compiled using gene names associated with constituent proteins previously identified in proteomic studies of PEX material.^2^^,^^21^^–^^32^ Finally, an additional previously published transcriptional data gene set was generated to reinvestigate differential expression of genes previously analyzed in gene transcription studies of PEX.^26^^–^^29^^,^^31^^,^^34^^,^^47^ Expression differences in each gene set were quantified using χ^2^ tests to compare the number of genes differentially expressed in the relevant gene set's predominant direction of differential expression, against the number of genes differentially expressed in the same direction within the complete dataset (see the Appendix for gene lists and accession resources).

### Histology, Immunohistochemistry, and Electron Microscopy

A histopathological analysis and immunohistochemical studies were performed using formalin-fixed and paraffin-embedded lens capsular tissue obtained at routine cataract surgery from one patient with PEX and one without PEX. Immunohistochemical staining for type 4 collagen (COL4) was performed using an automated immunohistochemistry platform (Ventana–Benchmark Ultra, Roche, Basel, Switzerland). Protease enzymatic antigen retrieval was performed for 8 minutes. The primary antibody was a mouse anti-COL4 IgG (CIV22) (Cell Marque, Rocklin, CA) raised against a human COL4 immunogen. Primary antibody titrations demonstrated an optimal dilution of 1:50. Incubation was performed at 36°C for 36 minutes. Additionally, 4 PEX and 4 non-PEX lens capsular samples were collected from patients undergoing cataract surgery were fixed in glutaraldehyde and subjected to transmission electron microscopy. Positive controls included 2 normal human lung parenchymal samples. All images were reviewed by a pathologist (S.K.).

## Results

### Samples and Demographics

Lens capsular epithelium samples from 64 participants yielded sufficient RNA of acceptable quality (RNA integrity numbers: 8.3 ± 0.6) for sequencing. A total of 3.3 × 10^9^ transcript reads were mapped, with a mean mapped read count (±2σ) of 5.2 × 10^7^ (±5.3 × 10^6^) per sample. One sample was excluded owing to low mapped read counts (i.e., >2σ from the mean [2.7 × 10^7^]; Supplementary Fig. S1). Samples from 25 PEX and 38 non-PEX participants were included in the primary differential expression analysis. Gender was similar between cohorts characterized by PEX status (*P* = 0.60) but the mean age was older in the PEX cohort (80.0 years vs. 75.2 years; *P* = 0.022) (Table 1). However, the PCA demonstrated that age was not associated with a major axis of variation in this study (Supplementary Fig. S2C). The same cohort was subcategorized based on the presence or absence of HTG. Within a secondary glaucoma analysis, 19 samples from participants with glaucoma were compared with 44 samples from participants with no history of glaucoma, with no gender or age differences observed between groups (Table 1; for individual sample demographic/phenotypic data, see the Appendix). All participants were of self-reported European ancestry.

**Table 1.** Sample Demographics According to Pseudoexfoliation and Glaucoma Status

	PEX		Non-PEX		*P* Value	
	PEX Glaucoma	PEX No Glaucoma	Non-PEX Glaucoma	Non-PEX No Glaucoma	PEX vs. Non-PEX	Glaucoma vs. No Glaucoma
Sample size (n)	8	17	11	27	*NA*	*NA*
Age (years)	81.4 (5.8)	79.4 (8.0)	73.7 (6.1)	75.8 (9.8)	0.022^*^	0.90
Gender (m:f [%male])	4:4 (50.0)	6:11 (35.2)	6:5 (54.5)	13:14 (48.1)	0.60	0.68

* *P* < 0.05.

Age and gender for the 63 participants with samples passing quality control, categorized according to PEX status or glaucoma status. Primary differential expression analysis compared participants based on presence of PEX (PEX vs. non-PEX). Secondary analysis of the same cohort was performed comparing participants based on presence of glaucoma (PEX glaucoma + non-PEX glaucoma vs. PEX no glaucoma + non-PEX no Comparisons were made comparing PEX vs. no-PEX, and glaucoma vs. no glaucoma. Age comparisons were performed using independent sample paired *t*-tests, and gender comparisons were performed using χ^2^ tests. Age is reported using mean and standard deviation.

N/A, not applicable.

### Principal Components Analysis

A PCA performed using raw transcript counts per gene across all samples demonstrated that >80% of the variance between samples was accounted for by the first 2 principal components (PCs) (Supplementary Fig. S2A). The major genes driving the top PCs were mitochondrially encoded genes (Supplementary Fig. S2B). Further PCA demonstrated that additional covariates, including age and gender contributed minimally to the observed variation between samples (Supplementary Fig. S2C).

### Transcriptional Overview

**Figure 1.**
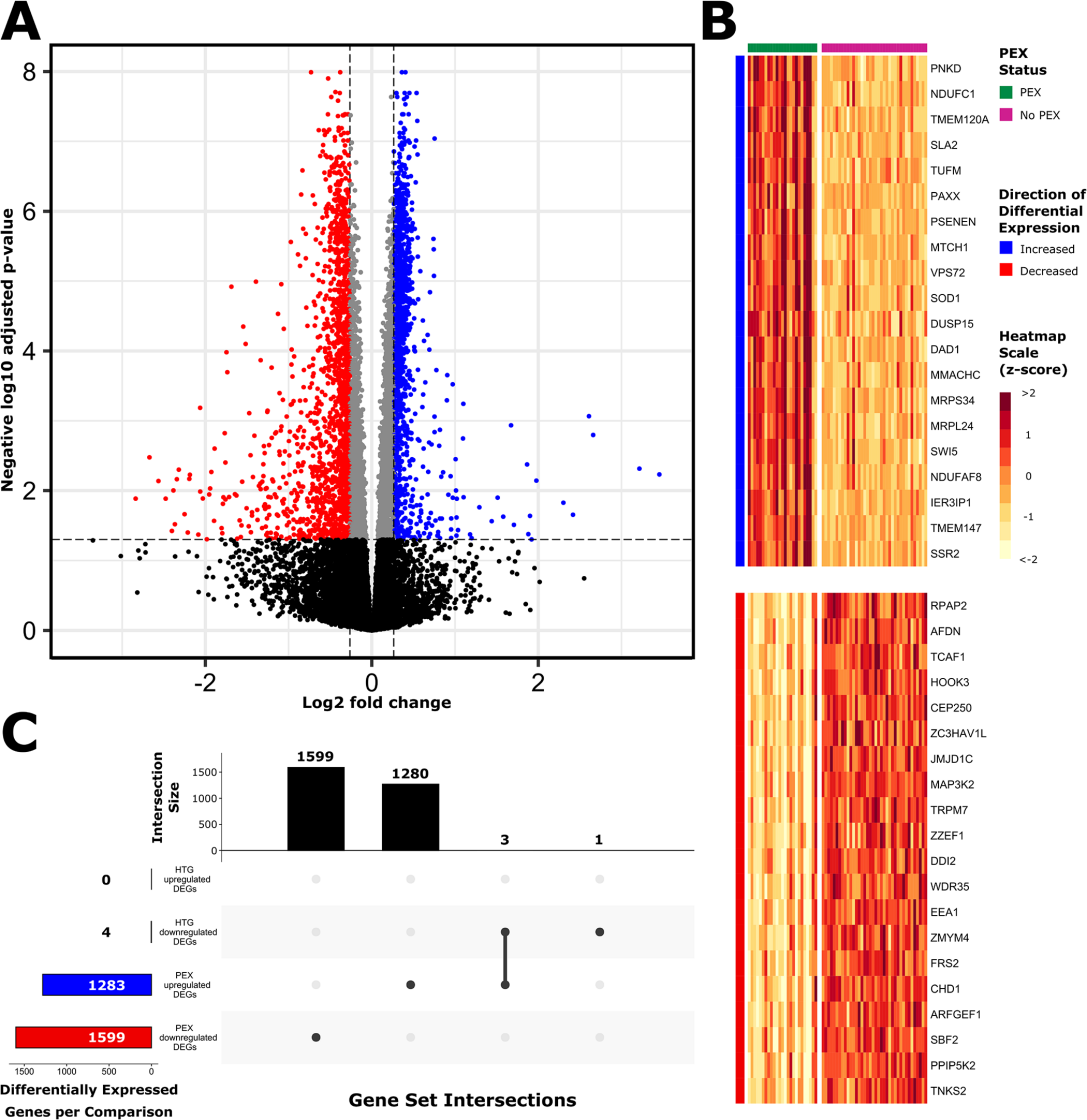
Differential gene expression profile. Genes differentially expressed in a PEX versus non-PEX comparison (PEX: *n* = 25; non-PEX: *n* = 38) are represented on a volcano plot (**A**), with DEGs defined by an adjusted *P* value of less than 0.05 (*horizontal dashed line*), and log_2_ fold expression change of ± log_2_(1.2) (*vertical dashed lines*). Upregulated and downregulated genes are indicated by blue and red dots, respectively. A corresponding heatmap demonstrates the 20 most significantly upregulated and downregulated genes (**B**). An UpSet plot demonstrates the absolute number of upregulated and downregulated genes in separate comparisons based on PEX status (i.e., PEX versus non-PEX) and glaucoma status (i.e., glaucoma vs. non-glaucoma) in the same cohort (**C**). A total of 2882 genes were differentially expressed according to PEX status. Four genes were differentially expressed according to glaucoma, only one of which was uniquely differentially expressed in this comparison.

From a total of 15,791 genes with measurable expression across all samples, 2882 genes demonstrated differential expression in the PEX versus non-PEX comparison (i.e., log_2_ fold change ± log_2_1.2; adjusted *P* < 0.05). Among the DEGs, 1283 were upregulated and 1599 were downregulated in PEX relative to non-PEX (Fig. 1A&B). Secondary differential expression analyses comparing glaucoma to non-glaucoma samples within the same cohort identified 4 DEGs, 3 of which were also differentially expressed in the PEX versus non-PEX analysis (*EGR2*, *FOSB*, and *EGR3*), and one of which was uniquely differentially expressed in the glaucoma versus non-glaucoma analysis (*USP6*; Fig. 1C). Furthermore, PCA a analysis demonstrated no axis of variation linked to glaucoma status, suggesting that neither glaucoma status nor topical ocular antihypertensive therapy were strong confounding factors, and that PEX was the major factor associated with differential gene expression in lens capsular epithelium in the current study.

To determine the consistency of gene expression profiles across samples characterized by the presence or absence of PEX, we used the *pheatmap* function with the argument *cluster_cols = TRUE* to group individual samples based upon their expression of the 40 most DEGs. Within this analysis, 78.3% of samples (55 of 63) clustered appropriately according to their phenotype (PEX or non-PEX). Although the remainder of samples demonstrated discordant clustering (8/63 [12.7%]) (Supplementary Fig. S3A&B), further PCA using the entire expression dataset (i.e., 15,791 genes with measurable expression and first 3 PCs) demonstrated appropriate phenotypic grouping of these eight samples (Supplementary Fig. S3C).

### Pathway Analysis

**Figure 2.**
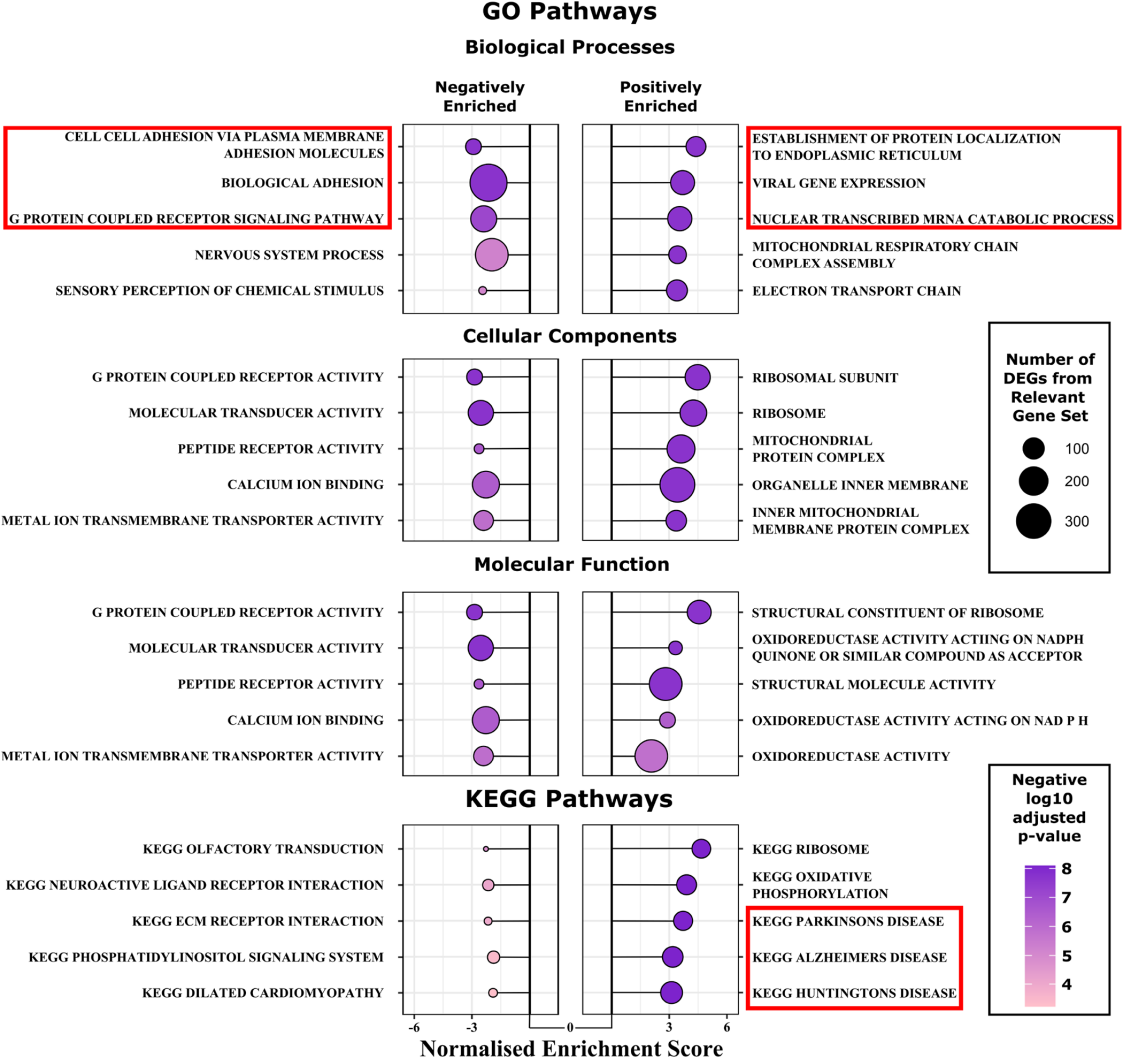
Pathways analysis. Enriched GO terms and KEGG pathways in PEX syndrome cases versus non-pseudoexfoliation controls (PEX: *n* = 25; non-PEX: *n* = 38). Positive and negative normalized enrichment scores indicate upregulated and downregulated pathways, respectively. GO terms are subclassified into Biological Processes, Cellular Components, and Molecular Function. Normalized enrichment scores quantify the directional enrichment value of a specific pathway while accounting for gene set size in the context of the expression dataset. Pathways are primarily ranked by adjusted *P* value, and secondarily ranked by the normalized enrichment score in pathways with identical adjusted *P* values. Point size represents the absolute number of genes from a pathway that were expressed differentially in the current study. Point color represents the adjusted *P* value attributed to the gene set enrichment of the relevant pathway. Specific pathways investigated further are indicated within *red boxes*.

All genes for which expression differences between PEX and non-PEX samples were associated with adjusted *P* value of less than 0.05 were subjected to gene set enrichment analysis using GO and KEGG gene sets to identify enriched pathways. From a total of 419 significantly enriched GO pathways (i.e., adjusted *P* < 0.05), 190 intercorrelated pathways were removed using the *Revigo* tool. This analysis identified an upregulation of genes associated with mitochondrial and ribosomal pathways in PEX relative to non-PEX. It additionally demonstrated a PEX-specific downregulation of genes involved in pathways associated with cellular adhesion and plasma membrane function (Fig. 2). Several unique results included positive enrichment of the GO *viral gene expression* pathway (GO:0019080), an annotation that includes genes demonstrated to be dysregulated in the context of viral infection, and KEGG pathways representing neurodegenerative diseases of the central nervous system (Parkinson's disease, Alzheimer's disease, and Huntington's disease). Individual genes from the most significantly enriched of these pathways were investigated further across samples.

### Positively Enriched Pathways

**Figure 3.**
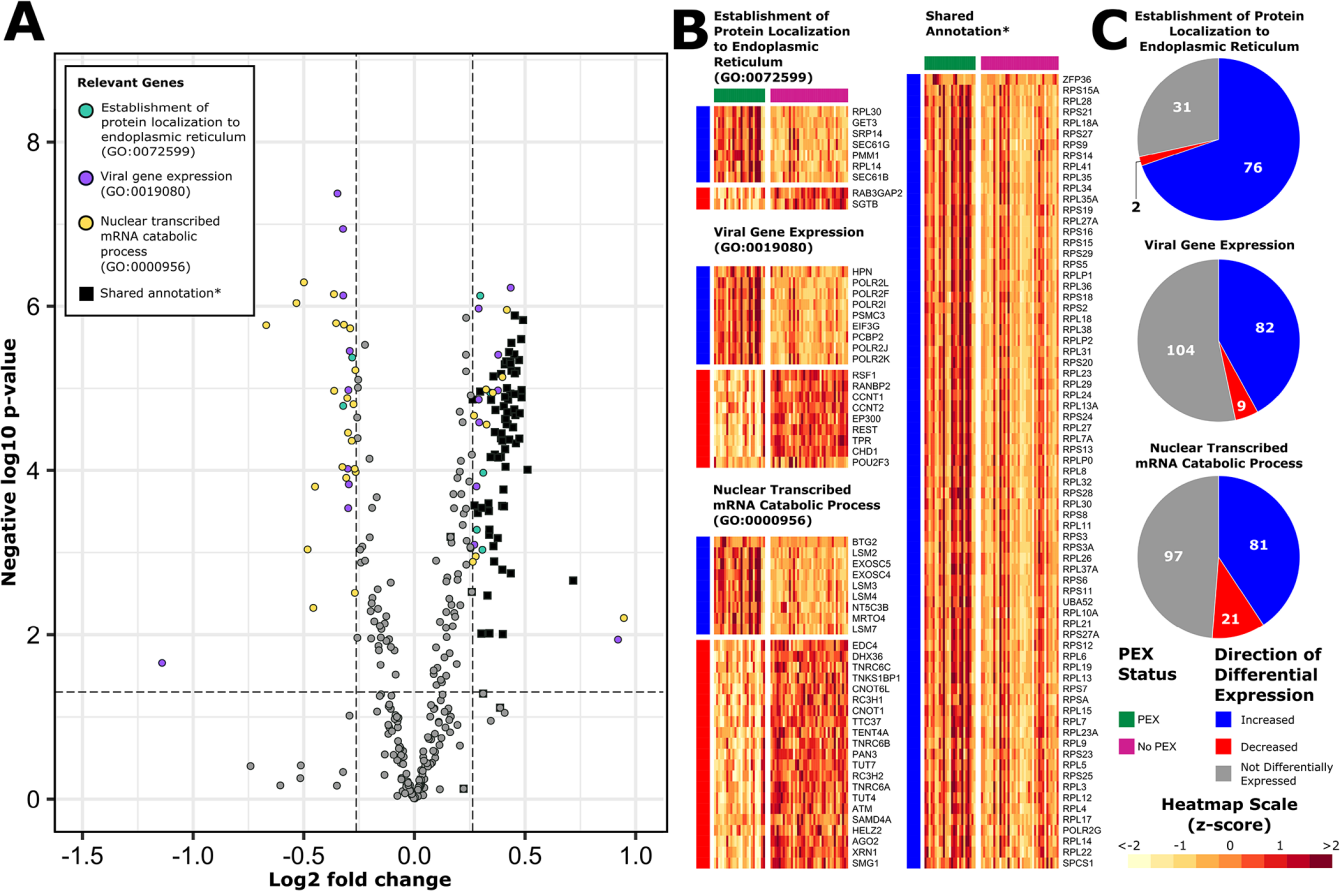
Positively enriched GO biological processes. DEGs from the most positively enriched “GO: Biological Processes” gene set annotations are represented on a volcano plot (**A**) and heatmaps (**B**). *DEGs that were common to the 3 gene sets are represented separately on a heatmap titled Shared Annotation. Pie charts demonstrate the proportions of genes with measurable transcripts from each of these pathways which were upregulated (*blue*), downregulated (*red*), or not differentially expressed (*grey*).

We performed a more detailed investigation of the individual genes contributing to the 3 most positively enriched pathways (Fig. 3). Despite gene set pruning with the *Revigo* tool, many DEGs were shared between these pathways. Common to the 3 most positively enriched pathways were multiple genes which encode ribosomal subunits. Upregulated DEGs unique to the *establishment of protein localization to endoplasmic reticulum* pathway (GO:0072599) included genes encoding transmembrane channels such as SEC61B and SEC61G, which facilitate trafficking of nascent proteins to and from the endoplasmic reticulum for post-translational modification, and enzymes such as PMM1, which directly catalyze post-translational protein modification. The *viral gene expression* pathway (GO:0019080) largely constitutes genes encoding cellular machinery required for production of proteins and nucleic acids, including those of viral origin. Accordingly, 39.9% of these genes encode ribosomal proteins which are transcriptionally upregulated during viral infection to facilitate viral replication.^48^ Various DEGs unique to this gene set include those encoding subunits of RNA polymerase II (*POLR2I*, *POLR2J*, *POLR2K*, *POLR2F*, *and POLR2L*), a host protein essential for transcription, and also demonstrated to be integral to replication of RNA viruses.^49^ The *nuclear transcribed mRNA catabolic process* pathway (GO:0000956) consists of genes encoding proteins involved in the nonsense-mediated decay of transcripts carrying premature stop codons products. DEGs unique to this pathway include those encoding components of the exosome (*EXOSC4* and *EXOSC5*) and several RNA-binding proteins (*LSM2*, *LSM3*, *LSM4*, and *LSM3*).

### Negatively Enriched Pathways

**Figure 4.**
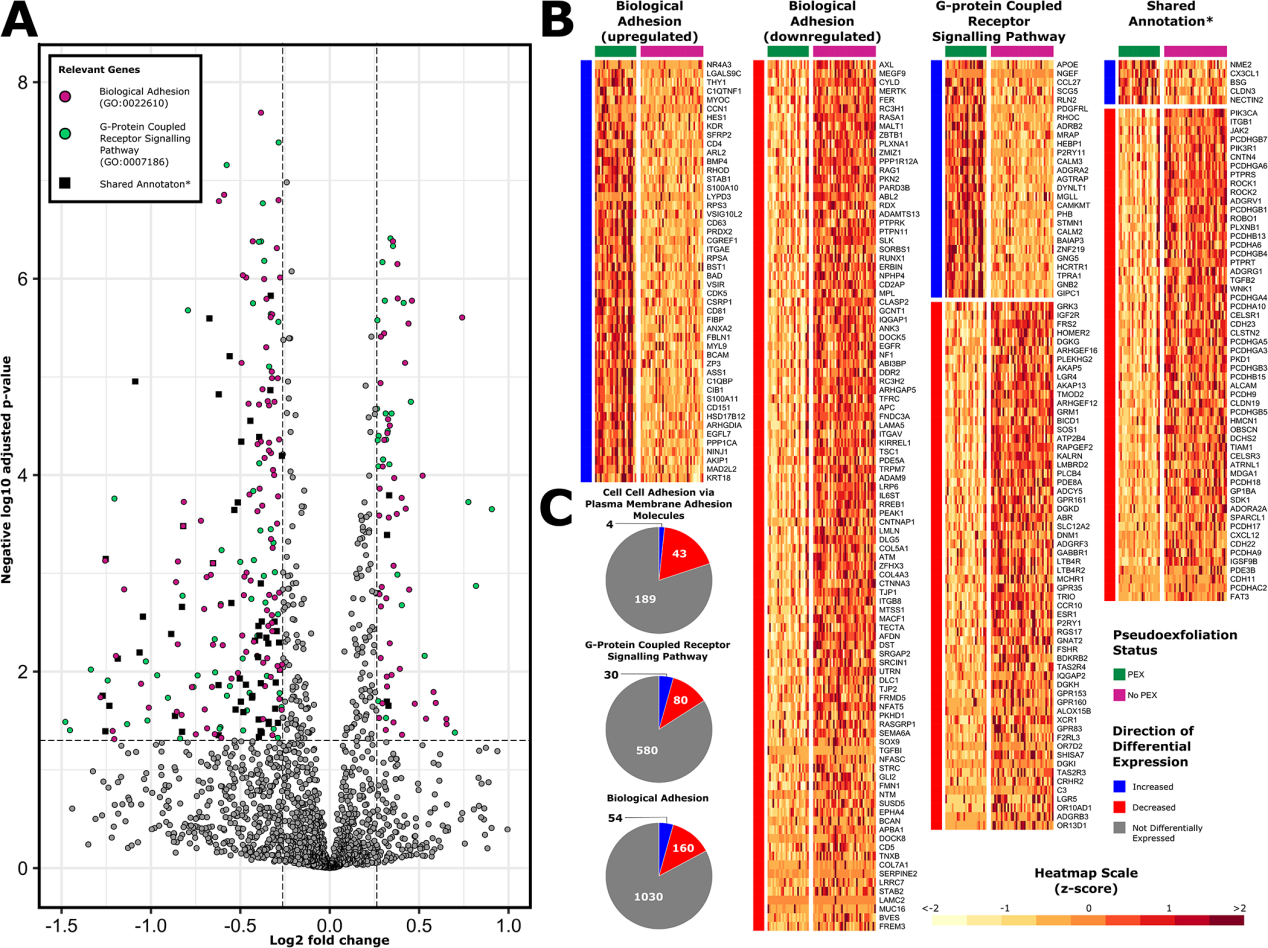
Negatively enriched GO biological processes. DEGs from the most negatively enriched ‘GO: Biological Processes’ gene set annotations are represented on this volcano plot (**A**) and heatmaps (**B**). DEGs which were common to the 3 gene sets are represented separately on a heatmap titled Shared Annotation*. Pie charts demonstrate the proportions of genes with measurable transcripts from each of these pathways, which were upregulated (*blue*), downregulated (*red*), or not differentially expressed (*gray*).

The 3 most negatively enriched GO pathways involved plasma membrane processes including cell adhesion and G-protein–coupled cell signaling (Fig. 4). Again, despite pruning of overlapping pathways using the *Revigo* tool, many DEGs were shared between 2 or more of these pathways. Furthermore, all DEGs within the *cell–cell adhesion via plasma adhesion molecules* pathway (GO:0098742) were encompassed within the 2 other investigated pathways. The larger *biological adhesion* pathway (GO:0022610) incorporates a set of genes involved in intercellular connection and cell matrix adhesion. Downregulated genes within this set include several encoding collagen subunits (*COL4A3*, *COL5A1*, and *COL7A1*). Genes encoding several cadherins and protocadherins, which are important cell adhesion proteins, were downregulated in both of these pathways. Loss of these proteins may result in decreased adhesion of cells within their tissue matrix. Negative enrichment of the *G-protein coupled receptor signalling* pathway (GO:0007186) suggests that PEX lens capsular epithelium may have either decreased activation or decreased sensitivity in the setting of specific extracellular signals.

### Enriched KEGG pathways

**Figure 5.**
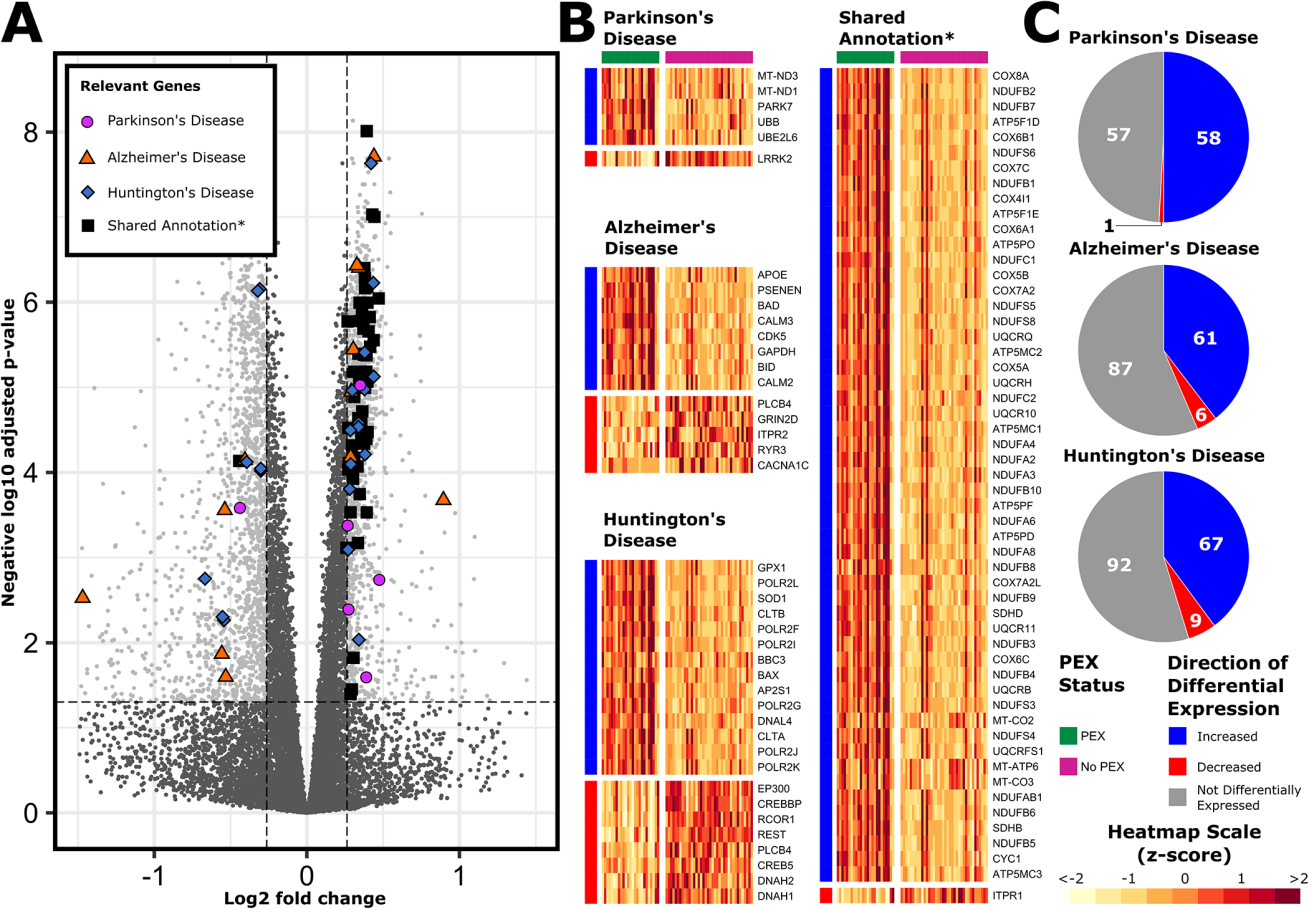
Enriched KEGG disease pathway genes. After ribosome and oxidative phosphorylation, the 3 most positively enriched KEGG pathways were the disease annotated pathways Parkinson's disease, Alzheimer's disease, and Huntington's disease. DEGs from these 3 gene sets are represented on this volcano plot (**A**) and heatmaps (**B**). *DEGs that were common to the 3 gene sets are represented separately on a heatmap entitled Shared Annotation. Pie charts demonstrate the proportions of genes with measurable transcripts from each of these pathways that were upregulated (*blue*), downregulated (*red*), or not differentially expressed (*gray*).

Distinctive pathways highlighted through KEGG analysis included 3 pathways associated with neurodegenerative conditions, namely Parkinson's disease, Alzheimer's disease, and Huntington's disease. Like PEX, Parkinson's disease and Alzheimer's disease are characterized by extracellular aggregation of pathological proteins. Apolipoprotein E (*APOE*), encoding a major lipid transport molecule within the central nervous system, and variants that are associated strongly with Alzheimer's disease risk, was upregulated in this dataset. APOE has also been identified as a component of PEX material.^32^ On further analysis of individual DEGs from each of these gene sets, it was observed that most upregulated genes were shared between pathways (Fig. 5). Much of the signal driving the positive enrichment of these 3 disease gene sets was the result of general upregulation of groups of chromosomal genes involved in the respiratory transport chain (*COX*, *NDUF*, *ATP5*, and *UQCR* subunit genes), and mitochondrially encoded genes (*MT-CO2*, *MT-CO3*, and *MT-ATP6*).

### Class-Specific Gene Set Analysis

Recognizing common themes of increased ribosomal protein and mitochondrial respiratory transport chain gene expression, and decreased plasma membrane adhesion protein expression, we sought to investigate differential expression characteristics of specific HUGO gene classes within this sample. Also using HUGO gene sets, we investigated differential expression of other gene classes previously implicated in studies investigating the pathophysiology of PEX. The ribosomal proteins, mitochondrial respiratory chain complex, and cell adhesion molecules were the most significantly perturbed gene classes analyzed (Table 2).

**Table 2.** Gene Classes

Gene Set	Measured Genes	Upregulated	Downregulated	Dominant Direction	*P* Value
Whole sample	15,791	1283	1599		
Ribosomal genes	168	111	0	Up	**<0.0001** ^***^
Mitochondrial respiratory chain complex	110	58	0	Up	**<0.0001** ^***^
Mitochondrial genomic genes	13	5	0	Up	**0.003** ^**^
Cell adhesion molecules	485	16	70	Down	**0.006** ^**^
Protocadherins	61	0	19	Down	**<0.0001** ^***^
Cadherins	26	0	4	Down	0.43
Ig superfamily	360	15	42	Down	0.39
Integrins	25	1	3	Down	0.78
Mucins	13	0	2	Down	0.58
Selectins	0	*NA*	*NA*	*NA*	*NA*
Collagens	38	0	8	Down	0.055
Ubiquitin-specific proteases	49	0	11	Down	**0.01** ^*^
Unfolded protein response genes	110	13	3	Up	0.13
TGFB family	7	0	1	Down	0.75
TGFB genes	3	0	1	Down	0.27
LTBP genes	4	*NA*	*NA*	*NA*	*NA*
Matrix metallopeptidase family genes	19	*NA*	*NA*	*NA*	*NA*
Matrix metallopeptidases	16	*NA*	*NA*	*NA*	*NA*
TIMPs	3	*NA*	*NA*	*NA*	*NA*
Other genes					
Autophagy genes	32	5	4	Up	0.79
Interleukins	20	1	0	Up	0.68
Crystallins	15	2	2	Neither	*NA*
Fibrillins	3	*NA*	*NA*	*NA*	*NA*

* *P* < 0.05.

** *P* < 0.01.

*** *P* < 0.001.

Specific gene classes investigated in the current dataset were analyzed to determine enrichment of DEGs. Expressed genes were defined as the number of protein coding genes with expression characteristics exceeding Cook's cutoff (*DESeq2*). Representative *P* values were derived using χ^2^ tests comparing the number of genes within each gene set which were differentially expressed in the relevant gene set's predominant direction of differential expression, against the number of genes differentially expressed in the same direction within the complete dataset.

### Ribosomal Protein Coding Genes

**Figure 6.**
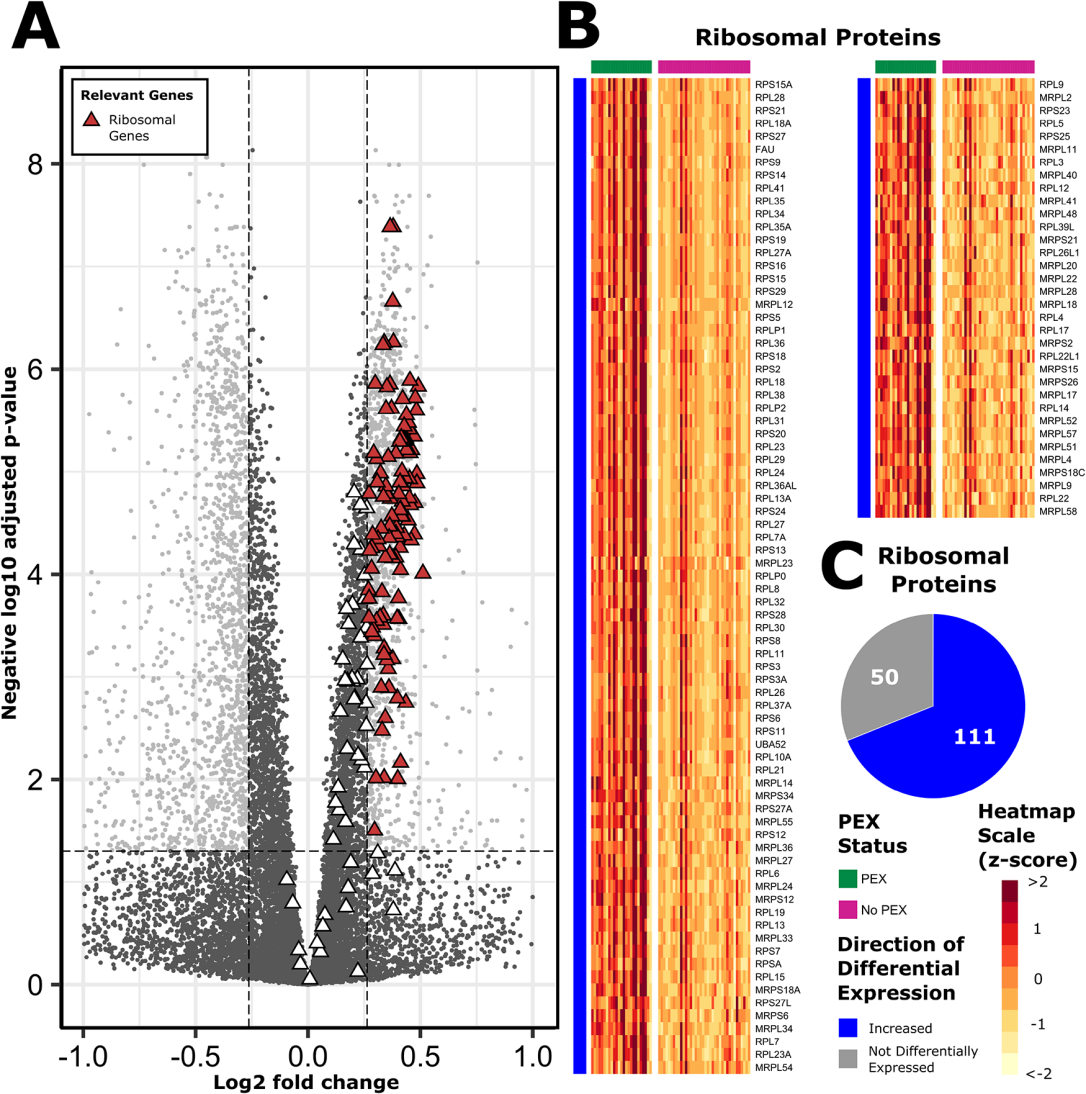
Ribosomal genes. Ribosomal genes which were differentially expressed in PEX cases vs. non-PEX controls (PEX: *n* = 25; non-PEX: *n* = 38) are represented in a volcano plot (**A**). DEGs defined by a log_2_ fold change of ±log_2_(1.2) (*vertical dashed lines*) and an adjusted *P* value of less than 0.05 (*horizontal dashed line*) are represented by colored points. Corresponding heatmaps demonstrate normalized individual expression (z-scores) of the same genes from the ribosomal genes set (**B**). Pie charts demonstrate the absolute number and proportions of genes according to their differential expression characteristics (**C**); *blue*, upregulated; *red,* downregulated; *gray,* no differential expression.

Our study identified an upregulation of genes encoding the small (S), large (L), and mitochondrial (M) ribosomal subunits which constitute the structural basis of the ribosomes. Of these genes, 111 out of 168 (66.1%) were upregulated, and none were downregulated in PEX samples (*P* < 0.0001) (Fig. 6).

### Mitochondrial Respiratory Chain Complex Genes

**Figure 7.**
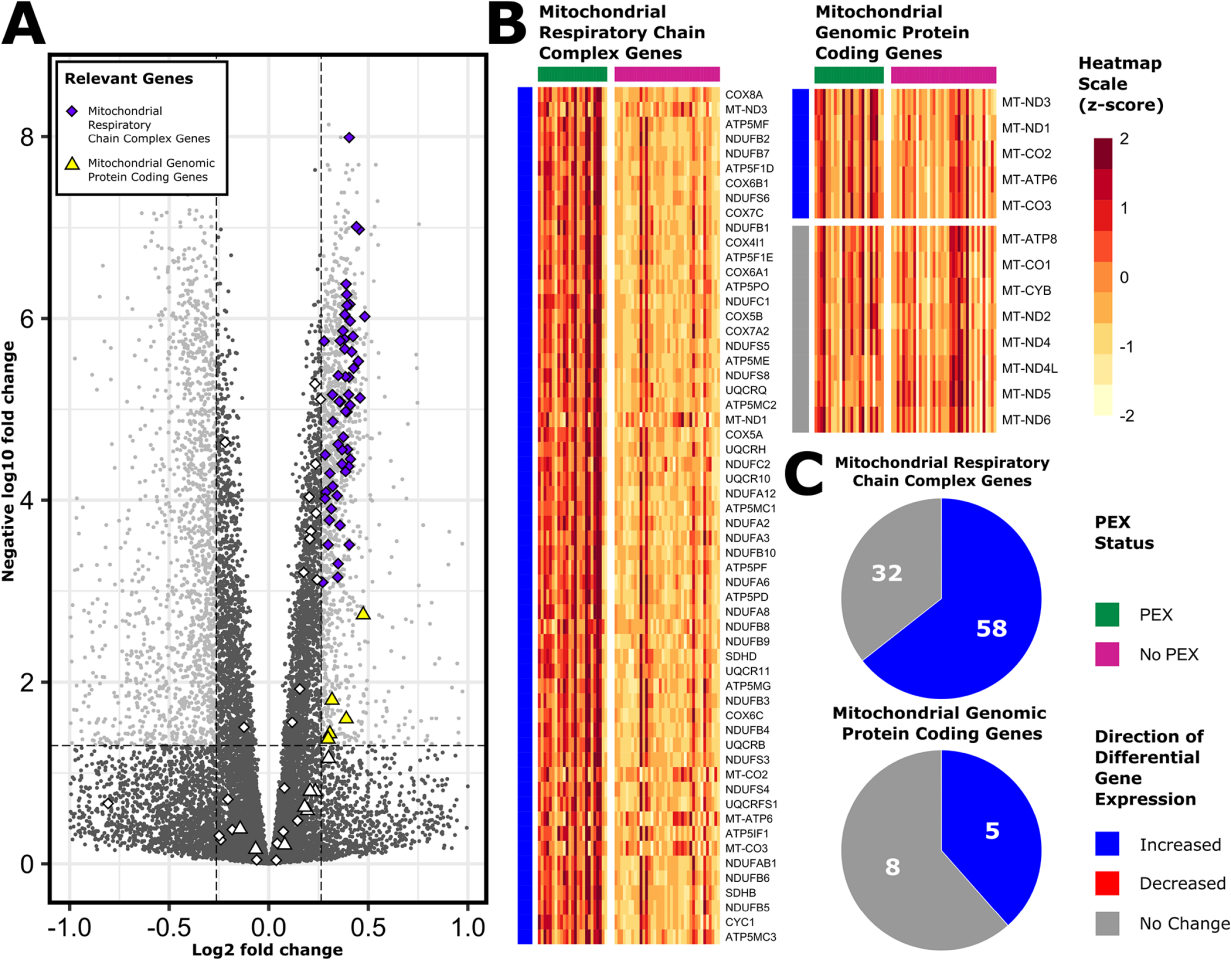
Chromosomal and mitochondrial genes involved in the mitochondrial respiratory chain complex. Genes involved in the mitochondrial respiratory chain complex which were differentially expressed in PEX cases versus non-PEX controls are represented in a volcano plot (**A**). DEGs defined by a log_2_-fold change of ±log_2_(1.2) (*vertical dashed lines*) and an adjusted *P* value of less than 0.05 (*horizontal dashed line*) are represented by colored points. Mitochondrial protein coding genes encoded by the mitochondrial genome (representing a subgroup of these genes) are represented by square points. Corresponding heatmaps demonstrate normalized individual expression (z-scores) of the same genes from the mitochondrial respiratory chain complex genes set (excluding mitochondrial protein coding genes) and the mitochondrial genomic protein coding genes set, respectively (**B**). Pie charts demonstrate the absolute number and proportions of genes from both gene sets according to their differential expression characteristics (**C**); *blue*, upregulated; *red*, downregulated; *gray*, no differential expression.

This study also observed a generalized upregulation of genes implicated in the mitochondrial respiratory chain complex (*P* < 0.0001). A total of 58 of the 97 genes encoding components of the mitochondrial respiratory chain complex (59.8%) were expressed differentially, all of which were upregulated (Fig. 7). This gene set contains both chromosomal and mitochondrial genomic genes that encode mitochondrial respiratory chain subunits, including cytochrome c oxidase, ATP synthase, nicotinamide adenine dinucleotide dehydrogenase, and ubiquinol cytochrome c reductase. Each of these enzymes is involved in cellular energy production through the process of oxidative phosphorylation. Furthermore, mitochondrial genomic genes—a gene list constituting all mitochondrially encoded protein coding genes—were also enriched, with 5 of the 13 genes upregulated and none downregulated (*P* = 0.003).

### Cell Surface Adhesion Molecule Genes

To further investigate the negatively enriched signals associated with processes underlying cellular adhesion, we investigated the differential expression profiles of 6 major groups of cell adhesion molecules (Supplementary Fig. S4). Collectively, cell adhesion molecules were downregulated (*P* = 0.006). The major subclass contributing to this signal was the protocadherins with 19 of 61 genes (31.1%) with measurable transcripts downregulated and none upregulated (*P* < 0.0001).

### TGFB and Latent TGFB Binding Protein Genes

The dysregulation of transforming growth factor beta proteins (TGFB) and latent transforming growth factor beta proteins (LTBP) has been implicated in previous studies of PEX. The upregulation of *TGFB2* was observed in the current study. However, no other transforming growth factor family members including *TGFB1*, *LTBP1*, and *LTBP2* were differentially expressed (Supplementary Fig. S5).

### Genes Encoding Matrix Metallopeptidases and Tissue Inhibitors of Matrix Metallopeptidases

Dysregulation of matrix metallopeptidases and their inhibitors, tissue inhibitors of matrix metallopeptidases (TIMPs), have been highlighted as potential contributory factors to the accumulation of extracellular material in PEX. No matrix metallopeptidases or TIMPs were differentially expressed in the current study (Supplementary Fig. S5).

### Unfolded Protein Response and Ubiquitin Specific Protease Genes

It has been hypothesized that PEX may result from disorder in either the unfolded protein response or ubiquitin-specific proteases (USPs).^47^ These 2 systems respond to endoplasmic reticulum stress and aberrant protein folding uniquely. The unfolded protein response responds to endoplasmic reticulum stress by increasing chaperone production and protein refolding, reducing protein synthesis, and increasing proteasomal protein degradation to decrease the misfolded or unfolded protein load.^50^ In contrast, USPs remove excess or abnormal proteins which have been ubiquitin-tagged for proteasomal degradation. In support of this hypothesis, we observed that 11 of 49 USP genes with measurable expression were downregulated in the current study (Supplementary Fig. S6). In concordance with previous data, we observed upregulation of ubiquitin (log_2_ fold change: 0.27; adjusted *P* = 0.004), the major USP tagging protein. In contrast, only one unfolded protein response gene (*PPP1R15A*) was differentially expressed (upregulated).

### Collagens

The downregulation of several collagen genes was noted within negatively enriched GO pathways. To interrogate this finding further, we investigated the differential expression profile of all collagen genes. Although the collagens gene class was not significantly enriched (*P* = 0.055), 8 of the 38 collagen genes with measurable expression were differentially expressed, all of which were downregulated (Supplementary Fig. S7). Two of the most highly expressed collagens, types 4 and 5, were differentially expressed, with 3 of the 4 measurable COL4 subunits (*COL4A1*, *COL4A3*, and *COL4A*), and 2 of the 3 COL5 subunits (*COL5A1* and *COL5A2*) demonstrating downregulation. An immunohistochemical study demonstrated no discernable difference in COL4 staining within PEX capsules compared with non-PEX capsules (Supplementary Fig. S8).

### Autophagy

Impaired autophagy has also been implicated as a potential disease mechanism in PEX.^51^ We investigated the differential expression profile of autophagy genes in the current study, but the data did not demonstrate enrichment of an autophagy gene class (*P* = 0.79). However, differential expression of several autophagy genes was observed, with the upregulation of *ATG4D*, *ATG10*, *ATG101*, *GABARAP*, and *MAP1LC3A*, and the downregulation of *ATG2B*, *ATG16L2*, *RB1CC1*, and *SNX30* (Supplementary Fig. S9).

### Proinflammatory Cytokines

Given patho-etiological associations between elevated proinflammatory cytokines and fibrotic diseases, including pulmonary fibrosis and systemic sclerosis, cytokines have been hypothesized to play a causal role in PEX. Elevated concentrations of IL-6 and IL-8 have been identified in serum and aqueous humor samples collected from patients with PEX.^52^^,^^53^ As a gene class, interleukins were not enriched in the current study (*P* = 0.68). Furthermore, *IL6* was not measurably expressed in the current study, and *IL8* (*CXCL8*) was not differentially expressed (Supplementary Fig. S9). *IL34* was the only differentially expressed interleukin, and was upregulated in PEX. The implications of increased *IL34* expression are unclear. However, *IL34* is known to be upregulated in response to viral infection.^54^

### Crystallins and Fibrillins

Crystallins and fibrillins have been previously implicated in biomolecular studies of PEX. Notably, fibrillin 1 has been both demonstrated to be upregulated in PEX tissues, and to be a constituent of PEX material.^26^^,^^28^^,^^29^^,^^32^ No fibrillins were differentially expressed in the current study (Supplementary Fig. S9). Several crystallins, which are major constituents of the crystalline lens, have also been identified to exhibit dysregulation in PEX. Beta-crystallin A3 (*CRYBA1*) was demonstrated to be upregulated in a RNA microarray study of PEX lens capsules.^28^ Crystallin beta B1 (*CRYBB1*), crystallin beta B2 (*CRYBB2*), and crystallin gamma D (*CRYGD*) were in higher concentration in a recent proteomic study of the aqueous humor of patients with PEX.^33^ The current study demonstrated differential expression of several crystallin genes, with upregulation of *CRYBB2* and *CRYGS*, and downregulation of *CRYBG3* and *CRYBG1* (Supplementary Fig. S9).

### Previous Protein and Gene Expression Data

We investigated the differential expression of genes which encode protein products identified in PEX material and genes that have demonstrated differential expression in PEX biospecimens. Among 74 genes encoding proteins previously identified in protein or proteomic studies of PEX, *APOE*, *CRYBB2*, *BFSP1*, *BFSP2*, *MYL6*, *FTH1*, *CRYAB*, and *SDC2* were upregulated (Supplementary Fig. S10). Among 30 genes demonstrating differential expression in previous transcription studies of PEX, only the upregulation of ubiquitin was concordantly differentially expressed (upregulated) in the current dataset (Supplementary Fig. S11).

### Disease-Associated Genes

Potential explanations for an association between PEX and its pathological ocular sequelae (cataract, zonular laxity, and HTG) were explored by investigating the differential expression profiles of genes implicated in Mendelian forms of disease (cataract, HTG, zonular instability [ectopia lentis]) or through GWAS (PEX). From a total of 386 investigated Mendelian cataract-associated genes, 323 were measurably expressed. Of these, 26 were upregulated and 42 were downregulated (Supplementary Fig. S12). Among the upregulated Mendelian cataract disease-associated genes were several crystallins (*CRYAB* and *CRYBB2*) and mitochondrial encoded genes (*MT-ATP6*, *MT-ND1*, and *MT-CO2*). Several COL4 subunit genes (*COL4A1*, *COL4A4*) were among the downregulated genes. Using a list of genes with single-nucleotide variants associated with PEX in GWAS, we investigated the expression profile of those genes in the lens capsular epithelium.^10^^–^^20^ From a total of 35 investigated PEX genes, all of which were measurably expressed, 2 were upregulated and 3 downregulated in PEX samples (Supplementary Fig. S13). Differentially expressed PEX GWAS loci associated genes included *APOE* and *POMP* which were upregulated, and *CACNA1A*, *SEMA6A*, and *FMN1* which were downregulated. Despite previous experimental data demonstrating differential expression of *LOXL1*^30^^,^^54^ and *CYP39A1*^20^ in various ocular tissues collected from patients with PEX, neither of these genes were differentially expressed in the current study. We investigated the differential expression profiles of Mendelian glaucoma-associated genes. From 21 glaucoma disease-associated genes with measurable expression, 2 were upregulated (*MYOC* and *FOXE3*) and five were downregulated (*SH3PXD2B*, *NF1*, *SBF2*, *ADAMTS17*, and *FOXC1*; Supplementary Fig. S13). An additional glaucoma gene list was generated using protein-coding genes collocated with POAG associated single-nucleotide variants. From a total of 169 collocated genes, 122 were protein coding genes. Six of these genes were upregulated and 21 were downregulated (Supplementary Fig. S14). Attempting to investigate for a possible transcriptional association between genes associated with zonular instability, we interrogated the current dataset to determine the differential expression profile of genes associated with Mendelian forms of ectopia lentis (Supplementary Fig. S13). Of 30 ectopia lentis disease-associated genes with measurable expression, 3 were upregulated (*MMACHC*, *PRDX1*, and *FOXE3*) and 3 were downregulated (*TGFB2*, *ADAMTS17*, and *FOXC1*; Supplementary Fig. S13).

### Histology and Electron Microscopy

**Figure 8.**
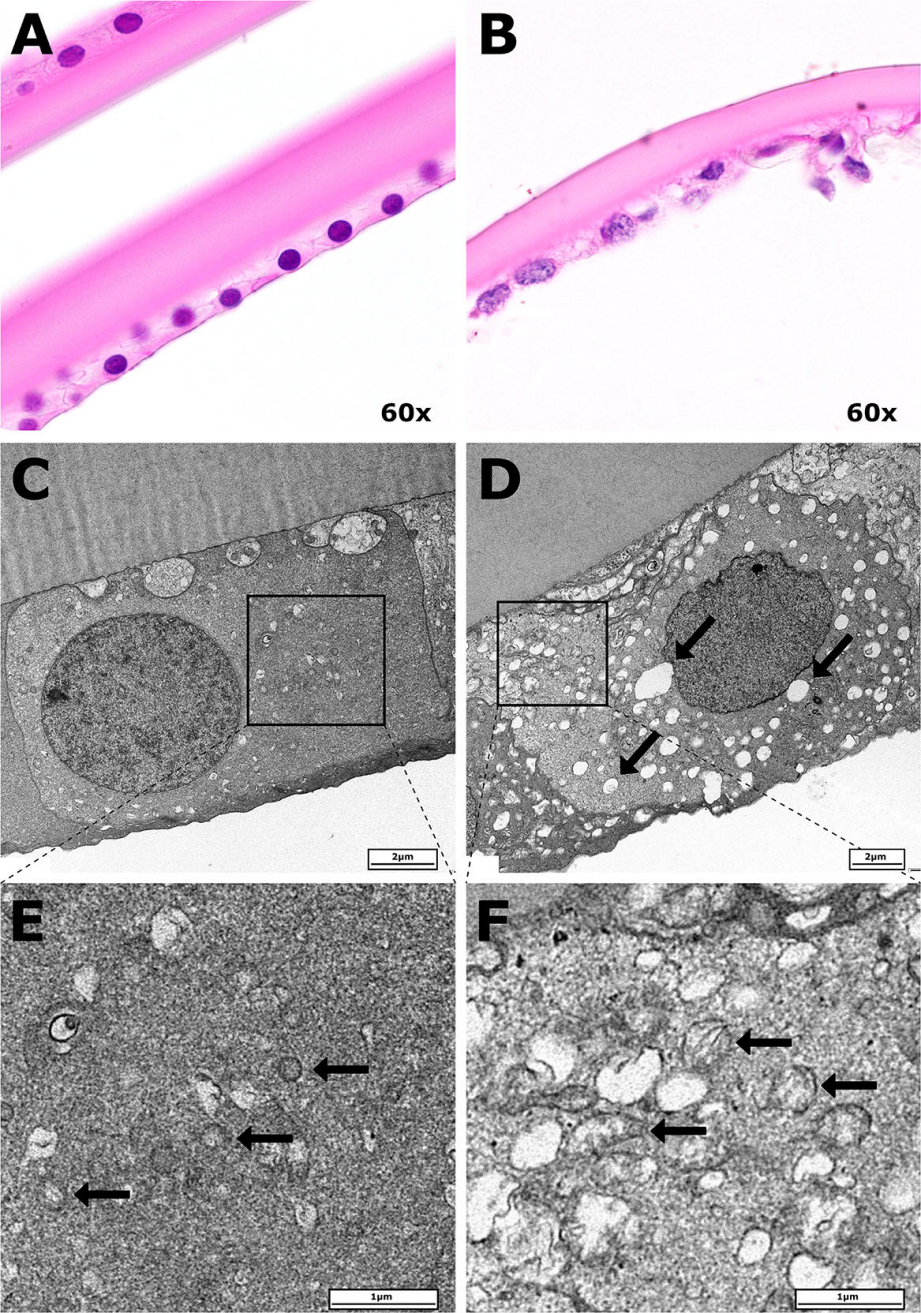
Lens capsule histology and electron microscopy. Hematoxylin and eosin staining of the lens capsule at 60× magnification demonstrates a single layer epithelium adherent to a basement membrane in a normal (non-PEX) specimen (**A**) and a PEX specimen in which epithelial cells are loosely adherent to the basement membrane (**B**). Nuclei within the PEX specimen demonstrate chromatin condensation and reactive-type nuclear atypia. Electron microscopy demonstrates characteristic cuboidal epithelial cellular architecture in the non-PEX lens capsular epithelium. A PEX lens capsular epithelium sample by contrast, demonstrates wrinkling of the cellular membrane, an atypical reactive-type nucleus, and dilatation of the endoplasmic reticulum (*diagonal arrows*; **D**). In comparison with morphologically normal mitochondria (*horizontal arrows*) in the non-PEX capsular epithelium (**E**), mitochondria in the PEX capsular epithelium (**F**) are enlarged and more abundant.

Recognizing strong dysregulatory transcriptional signals from the mitochondrial respiratory transport chain, ribosomes, and cell adhesion molecules, we performed histopathological and electron microscopic examinations of lens capsules collected from further individuals with or without PEX. Hematoxylin and eosin staining demonstrated PEX lens capsular epithelial cells which had lost their characteristic cuboidal cellular architecture, with some cells detaching from the basement membrane (Figs. 8A, B). Electron microscopy further demonstrated mitochondria which were dilated and enlarged in PEX, with generalized dilatation of the endoplasmic reticulum (Figs. 8C, D, E, F).

## Discussion

To the best of our knowledge, this RNAseq study is the first to investigate the transcriptional architecture of PEX, a common yet incompletely understood systemic disease associated with severe and often permanent vision loss. Observations from the current study have led to improved understanding of PEX, and the present dataset will provide a valuable reference tool for future studies seeking to improve knowledge of PEX and its ocular and systemic complications.

In parallel to genetic investigations of PEX which have identified multiple disease-associated risk loci,^10^^–^^19^ biological studies have demonstrated that perturbations in physiological processes including oxidative phosphorylation,^56^^,^^57^ autophagic flux,^51^^,^^58^ and the unfolded protein response^47^ may contribute to the PEX disease process. Furthermore, latitudinal differences in PEX prevalence have led to hypotheses that environmental exposures, such as ultraviolet light, may contribute to the disease.^59^ Despite progress in elucidating these features, the etiology and mechanisms by which PEX results in its ocular and systemic manifestation remain unclear. We chose to investigate PEX using the lens capsular epithelium as a substrate for several reasons. Primarily, the lens capsular epithelium is in direct contact with the crystalline lens where cataract, an ocular consequence of PEX manifests. Secondarily, there is evidence from electron microscopy studies that the lens capsular epithelium is one of several tissues involved in the production of fibrillar PEX material.^1^^,^^37^^,^^38^ Finally, ex vivo lens capsular epithelial samples are readily collectable as capsulorhexis specimens during cataract surgery.

Several novel features were evident from our RNAseq analysis. Primarily, this study identified a strong signal for ribosomal biogenesis within the PEX lens capsular epithelium. Ribosomal biogenesis is a complex and energy-consuming process implicated in various disease processes, and is most studied in Mendelian diseases and cancer. Viral infection, which was highlighted through enrichment of the *viral gene expression* (GO:0019080) gene set in our pathway analysis, is another recognized cause of ribosomal biogenesis that has been proposed as a potential pathological trigger for PEX. Detorakis et al.^60^ investigated a possible viral etiology in PEX using surgical biospecimens, in which a higher abundance of herpes simplex viral DNA was demonstrated in a case-control design using PEX and non-PEX iris samples. Furthermore, a viral hypothesis has been suggested following an epidemiological report demonstrating a high prevalence of PEX in partners of PEX affected individuals,^61^ and case reports of early onset PEX following intraocular surgery.^62^^–^^66^ Our study revealed an upregulation of ribosomal and other host genes including RNA polymerases and *IL34* which are associated with viral infection, supporting that viruses may be integral to the pathophysiology of PEX. There may be benefits in investigating possible viral or infectious associations with PEX using contemporary sequencing methods.

The upregulation of genes involved in mitochondrial respiratory transport was also noted in PEX samples. The roles of mitochondria, reactive oxygen species, and oxidative stress have been long standing topics of discussion in PEX.^56^^,^^57^ Our results support existing data through demonstration that mitochondrial respiration is transcriptionally upregulated in PEX. Although the upregulation of oxidative phosphorylation was transcriptionally evident within PEX samples, the contribution of oxidative stress was unclear. Increased expression of superoxide dismutase (*SOD1*), encoding the main antioxidant enzyme in the lens capsular epithelium and crystalline lens,^67^, seems to suggest an active response to oxidative stress. However, SOD1, which is localized to the outer mitochondrial membrane, may also be physiologically upregulated in the context of increased mitochondrial metabolism.^68^ Furthermore, oxidative stress was not featured as enriched pathways within our pathway analysis. Consequently, it is possible that the observed upregulation of mitochondrial respiratory transport chain genes observed in this dataset may be a physiological response to increased physiological demand, possibly owing to energy intensive processes such as ribosomal biogenesis. Increased expression of mitochondrial genes was also the principal feature contributing to enrichment of the *KEGG Parkinson's Disease*, *KEGG Alzheimer's Disease*, and *KEGG Huntington's Disease* pathways. In these diseases, mitochondrial function is impeded through processes such as disordered mitophagy, oxidative stress, and impaired mitochondrial fusion–fission.^69^ In light of these shared differential expression profiles, additional work is required to further characterize the contribution of such mitochondrial processes to the pathophysiology of PEX.

Another observation within the current dataset was the downregulation of biological adhesion genes. The downregulation of biological adhesion in this study was observed as a decreased expression of adhesion molecules involved in a homophilic cellular connection and adherence to the basement membrane and to the extracellular matrix. These processes may account for several histological observations within the lens capsular epithelium including loss of cuboidal cellular architecture, and interrupted cellular adherence to the basement membrane.^70^ The loss of basement membrane connections, which may be associated with decreased expression of COL4 transcripts, might explain the clinical observation that PEX cataracts are often more easily removed through lens hydrodissection than non-PEX cataracts.^71^ The loss of biological adhesion within the capsular epithelium may contribute to the increased prevalence of cataracts reported in PEX. Cellular migration of lens epithelial cells, required for the maintenance of a transparent crystalline lens, is a process which requires cellular adhesion molecules including cadherins. Consequently, decreased cadherin expression, implicated in the pathogenesis of steroid-induced cataracts, may also contribute to cataract formation in PEX.^72^^,^^73^

Our results demonstrated some similarities with previous targeted gene expression data generated from PEX biospecimens. Most notably, we demonstrated a significant negative enrichment of USPs (*P* = 0.01), supporting the hypothesis that PEX may result from impaired protease activity, proteostasis, and consequent accumulation of proteins (i.e., PEX material).^47^ Disordered autophagy has also been hypothesized to contribute to the accumulation of misfolded proteins and toxic aggregates in PEX. Although the autophagy gene set was not enriched in the current study (*P* = 0.79), several autophagy genes were differentially expressed, providing further suggestion that disordered autophagy may be relevant to PEX.^58^ By contrast, we did not observe expression changes in extracellular matrix genes including *LTBP1*, *LTBP2*, *TIMP1*, and *TIMP2*, which have been demonstrated to exhibit differential expression in PEX tissues.^27^^,^^28^^,^^35^ Reasons for differences between these results and the current data are unclear. However, in contrast with the current study, which was performed using samples from patients with mild to moderate PEX, other studies used samples collected from post mortem specimens or patients requiring enucleation owing to end-stage PEX glaucoma.^27^^,^^28^^,^^35^ It is possible that these differences in differential expression are a consequence of disease severity. Our observation that *LOXL1* was not differentially expressed in PEX lens capsular epithelium was consistent with previous work by Khan et al.,^55^ who performed a real-time polymerase chain reaction experiment using PEX and non-PEX lens specimens. In contrast, the same study also demonstrated decreased LOXL1 expression in PEX glaucoma specimens. Another study by Schlötzer-Schrehardt et al.^30^ demonstrated increased expression of LOXL1 in early disease that was not evident in late disease. Several reasons for differing results between these studies and ours may include our absence or PEX severity stratification, different tissues and tissue collection methods, and differing sequencing platforms. Our study further demonstrated an increased expression of multiple genes that encode proteins identified in PEX material (*APOE*, *CRYBB2*, *BFSP1*, *BFSP2*, *MYL6*, *FTH1*, *CRYAB*, and *SDC2*). These findings provide additional evidence that the lens capsular epithelium may contribute to the production of circulating PEX material.

Our study also investigated ocular consequences of PEX including cataract, glaucoma, and zonular instability by analyzing differential expression of Mendelian genes implicated in heritable diseases associated with these disease features. Among the differentially expressed cataract-associated genes were the crystallin genes *CRYAB*, *CRYBB2*, and *CRYGS*, which encode subunits of the major protein constituents of the crystalline lens. Crystallins have been recognized to manifest complex molecular arrangements, and structural changes to these proteins are associated with cataract formation. It is possible that altered expression of these genes in the lens epithelium results in structural changes to the crystalline lens predisposing to cataract formation. Additionally, several mitochondrial cataract-associated genes (*MT-CO2*, *MT-ATP6*, and *MT-ND1*) were differentially expressed. Differential expression of these genes may be relevant to cataract formation through contribution to oxidative stress, or as a reflection of other causative metabolic processes occurring in the lens capsular epithelium. Associations between PEX and zonular instability were explored by investigating differential expression of ectopia lentis-associated genes. Although the prevailing hypothesis underlying zonular fiber fragility in this disease is that PEX material accumulating on zonular fibers results in proteolytic changes and degradation,^74^ the altered expression of specific proteins may also contribute to impaired maintenance of zonular stability. Pathological loss of functional variants within *TGFB2*^75^—a gene observed to be downregulated in PEX—can result in Loeys-Dietz syndrome (type 4), a disease that is associated with ectopia lentis. It is possible that decreased expression of TGFB2 may contribute to zonular instability in PEX. Several glaucoma disease–associated genes also demonstrated differential expression in the current study. Mechanisms by which altered expression of these genes might contribute to glaucoma in PEX are unclear. However, given *FOXE3*-associated glaucoma is a consequence of anterior segment dysgenesis, and *MYOC* contributes to glaucoma through accumulation of mutant MYOC protein aggregates, we suspect differential expression of these genes is unlikely to contribute to PEX glaucoma. We are limited in our ability to comment on the relationship between differentially expressed glaucoma GWAS genes and the pathophysiology of PEX, particularly given that many of the single nucleotide variants collocated with these genes occur in distant intergenic regions, and the effect sizes of these variants are small. Finally, we interrogated this dataset to determine the expression profile of genes associated with the risk of PEX through GWAS. Several of these genes were upregulated (*APOE* and *POMP*) and several downregulated (*CACNA1A*, *SEMA6A*, and *FMN1*), demonstrating they may be associated with the pathophysiological process or response to disease and supporting these genes as the responsible gene at the respective GWAS locus.

The current dataset has provided a valuable substrate to generate evidence supporting the established and novel pathophysiological features of PEX and pathological ocular sequelae. However, insights garnered from these data are limited to a single tissue involved in a systemic disease. It is, therefore, unclear whether the processes observed in the PEX lens capsular epithelium were due to intrinsic cellular dysfunction or were responses to change within the cellular environment, which could be due to basement membrane diffusion barrier resulting from passive deposition of PEX material rather than primary production of PEX material. Furthermore, the capsulorhexis samples used in this study represent a small region at the anterior surface of the lens, which may not manifest transcriptional changes occurring at the equatorial region where it is thought PEX material may be produced. Further research requires similar investigation of additional tissues such as the ciliary body, iris, trabecular meshwork, or the Tenon capsule, which are all implicated in the disease process. Valuable detail may also be provided by complementary techniques that measure DNA, other RNA species beyond mRNA, and further protein expression work to correlate with DEGs identified in this study and others. Our work was limited to protein coding, polyadenylated RNA species, and will miss potentially relevant details that may be ascertained through analysis of non-polyadenylated RNA species, nonprotein coding species, and small RNAs.

This study has further highlighted PEX as a transcriptionally and physiologically complex disease phenomenon. We anticipate that a multimodal approach using additional sequencing technologies may yield further insight into the pathophysiology of PEX, and lead to targeted therapies.

## Conclusions

The current study represents the first RNAseq transcriptional dataset of PEX, providing a rich data source of carefully controlled data to facilitate growth of knowledge about this complex disease. Using this data, we have highlighted novel disease pathways and contributed detail to existing mechanistic hypotheses. This dataset is available as a publicly accessible resource for future research (NCBI Sequence Read Archive accession number: PRJNA764775).

## Supplementary Material

Supplement 1

Supplement 2

Supplement 3

Supplement 4
