# Supplementary material for: RNA Sequencing of Lens Capsular Epithelium Implicates Novel Pathways in Pseudoexfoliation Syndrome
Source: Invest Ophthalmol Vis Sci. 2022 Mar 29;63(3):26. doi: 10.1167/iovs.63.3.26 (PMC8982629; doi:10.1167/iovs.63.3.26)
Supplement: Supplement 1 [file iovs-63-3-26_s001.pdf]

SUPPLEMENTARY MATERIALS

Supplementary Figures

- Supplementary Figure S1: Mapped Read Counts.
- Supplementary Figure S2: Principal Components Analysis
- Supplementary Figure S3: Clustering of Samples by Genes Expression Profiles
- Supplementary Figure S4: Cell Adhesion Molecule Genes
- Supplementary Figure S5: Transforming Growth Factor and Matrix Metalloproteinase Associated Genes
- Supplementary Figure S6: Ubiquitin Specific Proteases and Unfolded Protein Response Genes
- Supplementary Figure S7: Collagen Genes
- Supplementary Figure S8: Type 4 Collagen Immunohistochemistry
- Supplementary Figure S9: Other Relevant Gene Classes
- Supplementary Figure S10: Pseudoexfoliation Material Genes
- Supplementary Figure S11: Previously Identified Differentially Expressed Genes
- Supplementary Figure S12: Mendelian Cataract Disease-Associated Genes
- Supplementary Figure S13: Other Mendelian Disease-Associated Genes
- Supplementary Figure S14: Glaucoma GWAS Genes

APPENDICES

Gene List and Resources

- Supplementary Table S1: Analysed Gene Classes
- Supplementary Table S2: Disease-Associated Genes
- Supplementary Table S3: Genes Associated with Proteins Identified in Pseudoexfoliation Material
- Supplementary Table S4: Previously Identified Different Expressed Genes in Pseudoexfoliation Syndrome

Gene Expression Data

- Sample Data (attached as separate spreadsheet)
- Differentially Expressed Genes (attached as separate spreadsheet)
- Enriched Pathways (attached as separate spreadsheet)

SUPPLEMENTARY FIGURES

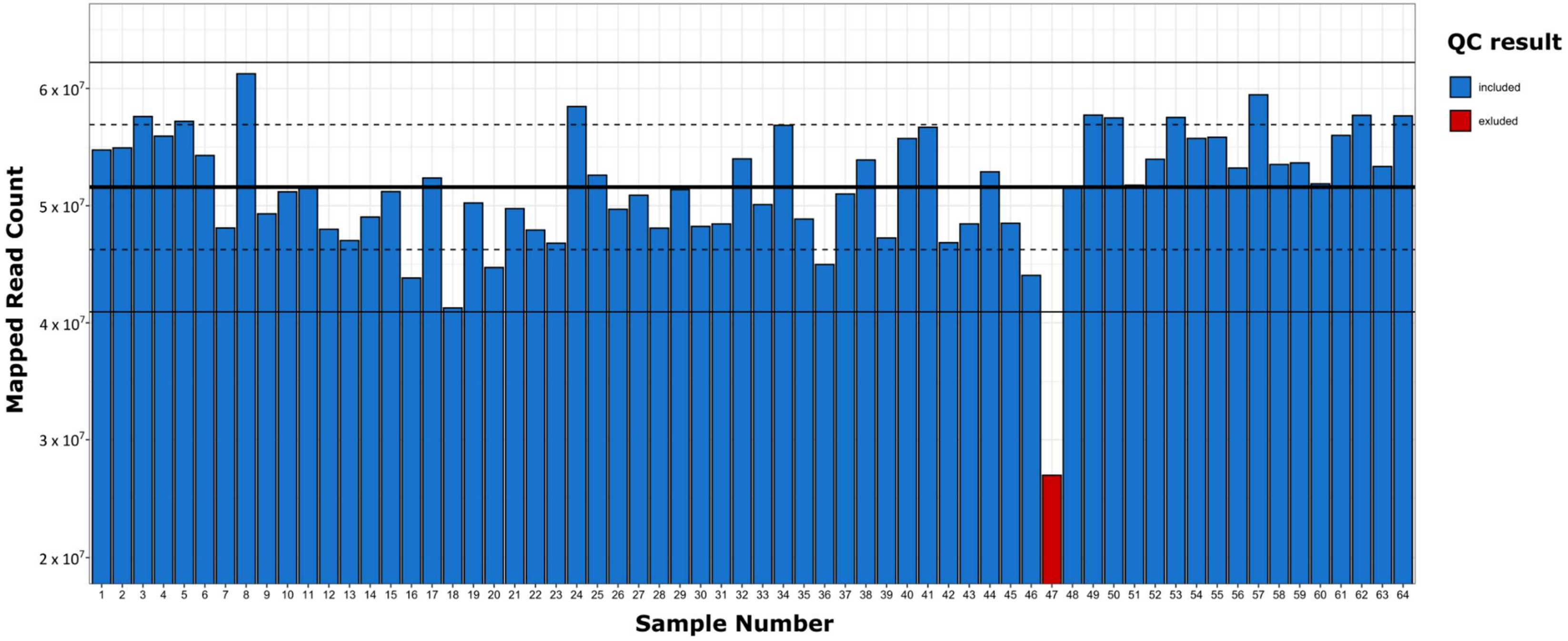

Supplementary Figure S1: Mapped Read Counts. A total of 3.3×10^{10} raw reads were generated from 64 lens capsular epithelium samples, with a mean mapped read count of 5.2×10^{7} (standard deviation = 5.3×10^{6}) counts per sample. Samples with a mapped read count of >2 standard deviations from the mean were excluded from downstream analysis. Included samples are indicated by blue bars and a single excluded sample (sample 47) is indicated by a red bar. Horizontal lines represent mean read count (thick continuous line), one standard deviation from the mean (dotted lines), and two standard deviations from the mean (thin continuous line); QC = Quality Control.

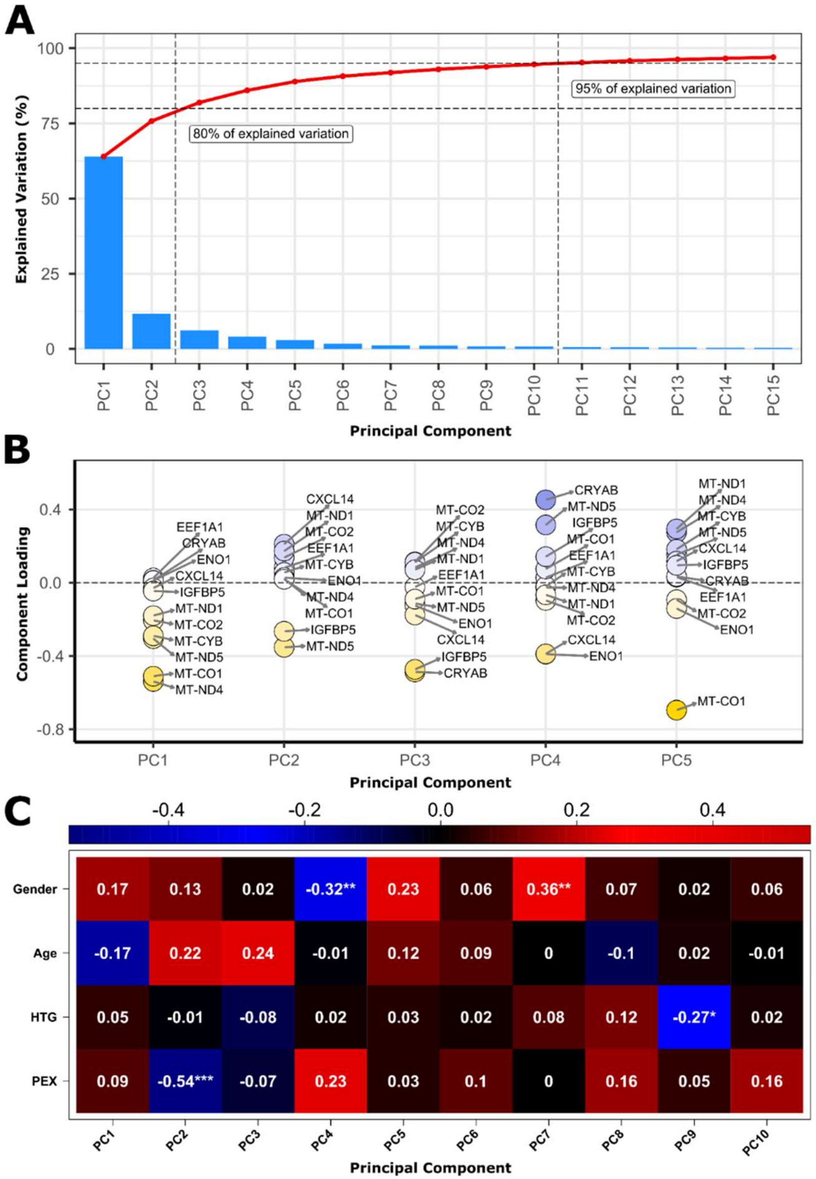

Supplementary Figure S2: Principal Components Analysis. A SCREEplot demonstrates the proportions of variance within the dataset are largely attributable to the first principal components, with 80% of the total variance being accounted for by the first two principal components, and 95% of the total variance attributable to the first ten principal components (A). A loadings plot demonstrates a high contribution of the variance within this dataset is the result of mitochondrial transcripts (B). An Eigencorplot demonstrates the relative contributions of gender, age, high-tension glaucoma status, and pseudoexfoliation disease status to the first ten principal components. HTG = high-tension glaucoma / glaucoma with elevated intraocular pressure; PEX = pseudoexfoliation disease; PC = principal component. * = p<0.05; ** = p < 0.01; *** = p<0.001 (Pearson's correlation).

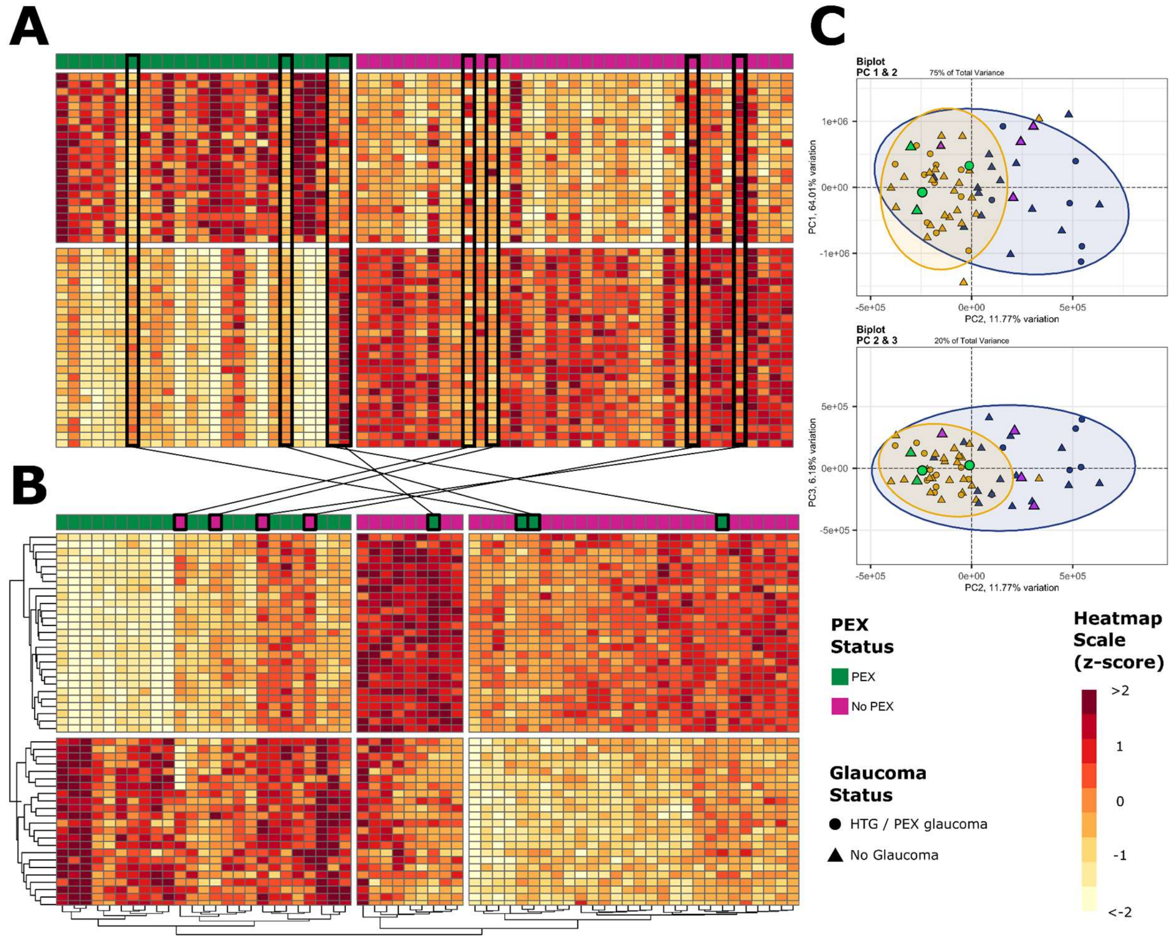

Supplementary Figure S3: Clustering of Samples by Genes Expression Profiles. Column based sorting was applied to visualise clustering of samples by phenotype in accordance with the 40 most differentially expressed genes. Columns in the top heatmap are grouped by the PEX and non-PEX phenotypes, with rows ordered by decreasing statistical significance (A). The order of samples displayed in this heatmap are consistent with all other heatmaps throughout this study. The bottom heatmap contains the same data with hierarchical clustering according to individual samples and genes (B). Samples clustering with the opposite disease phenotype are mapped back to the top heatmap. These apparently discordant samples were spatially mapped into biplots of the top three principal components from whole-sample principal components analysis, in which each clustered appropriately with other samples of the same pseudoexfoliation phenotype (C). Small blue circles and triangles: non-PEX specimens; yellow circles and triangles: PEX specimens; green circles and triangle: discordant PEX specimens; purple circles and triangles: discordant non-PEX specimens; Large shaded circles depict phenotypic clustering of samples defined by a confidence interval of 95% (yellow: PEX; blue: non-PEX); PC: principal component; PEX: pseudoexfoliation syndrome; HTG: high-tension glaucoma; PEX glaucoma: pseudoexfoliative glaucoma.

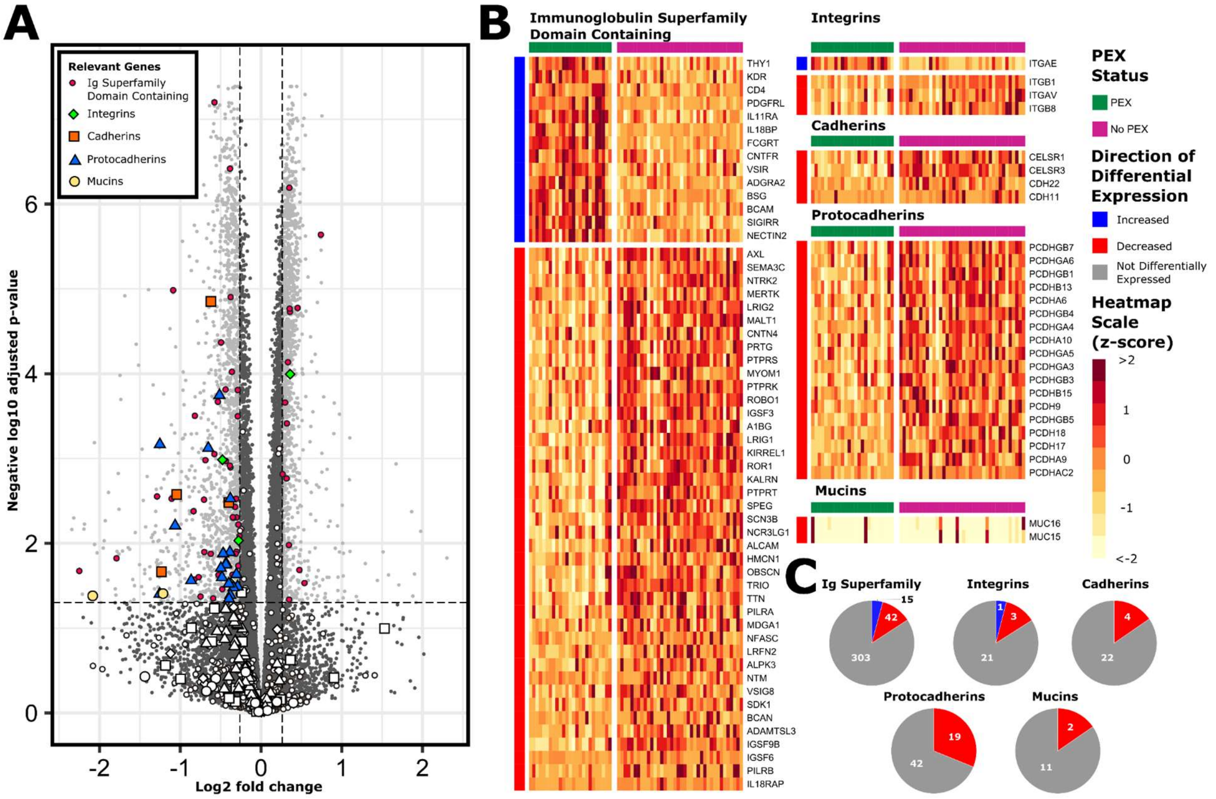

Supplementary Figure S4: Cell Adhesion Molecule Genes. Cell adhesion molecule genes which were differentially expressed in PEX cases vs. non-PEX controls are represented in a volcano plot (A). Differentially expressed genes defined by a log2 fold change of ± log2(1.2) (vertical dashed lines) and an adjusted p-value of < 0.05 (horizontal dashed line) are represented by coloured points. Corresponding heatmaps demonstrate normalised individual expression (z-scores) of the same genes from the ‘Immunoglobulin superfamily domain containing’, ‘Integrins’, ‘Cadherins’, ‘Protocadherins’, and ‘Mucins’ sets (B). The gene set for ‘Selectins’ is not represented as no genes from this list were differentially expressed. Pie charts demonstrate the absolute number and proportions of genes according to their differential expression characteristics (C); ‘blue’ = upregulated; ‘red’ = downregulated; ‘grey’ = no differential expression. Ig: Immunoglobulin; PEX: pseudoexfoliation syndrome.

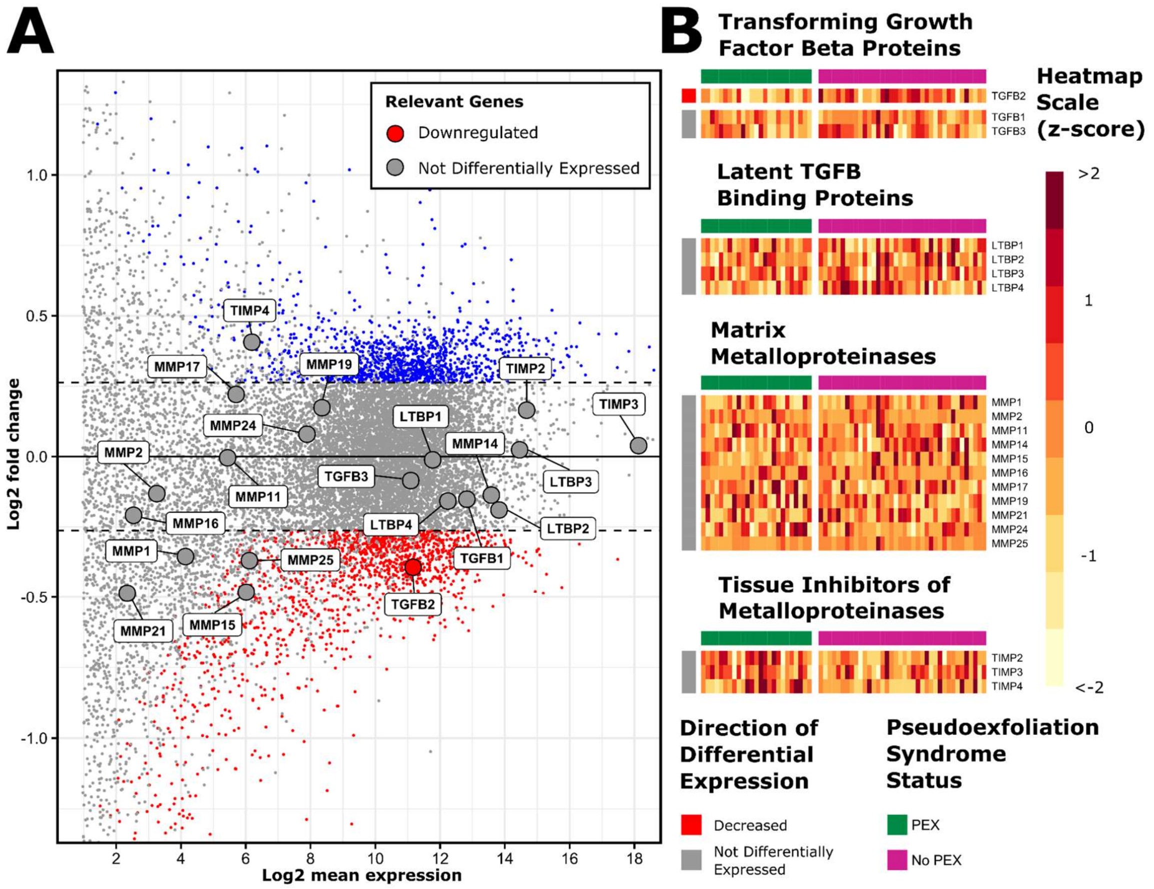

Supplementary Figure S5: Transforming Growth Factor and Matrix Metalloproteinase Associated Genes. An MAplot including all genes encoding transforming growth factors, latent transforming growth factor binding proteins, matrix metalloproteinases, and tissue inhibitors of matrix metalloproteinases demonstrates TGFB2 as the only differentially expressed (downregulated) gene from these four classes (A). Horizontal dashed lines represent the log2 fold change cutoff of $\pm$ log2(1.2). Differentially expressed genes (defined by a log2 fold change of $\pm$ log2(1.2) and padj < 0.05) are represented by blue (upregulated) and red (downregulated) points. Non-differentially expressed genes are represented by grey points. Individual gene expression per sample is represented as the normalised expression (z-score) per gene in class-specific heatmaps (B). PEX: pseudoexfoliation syndrome; TGFB: transforming growth factor beta; LTBP: latent transforming growth factor beta binding protein; MMP: matrix metalloproteinase; TIMP: tissue inhibitor of matrix metalloproteinases.

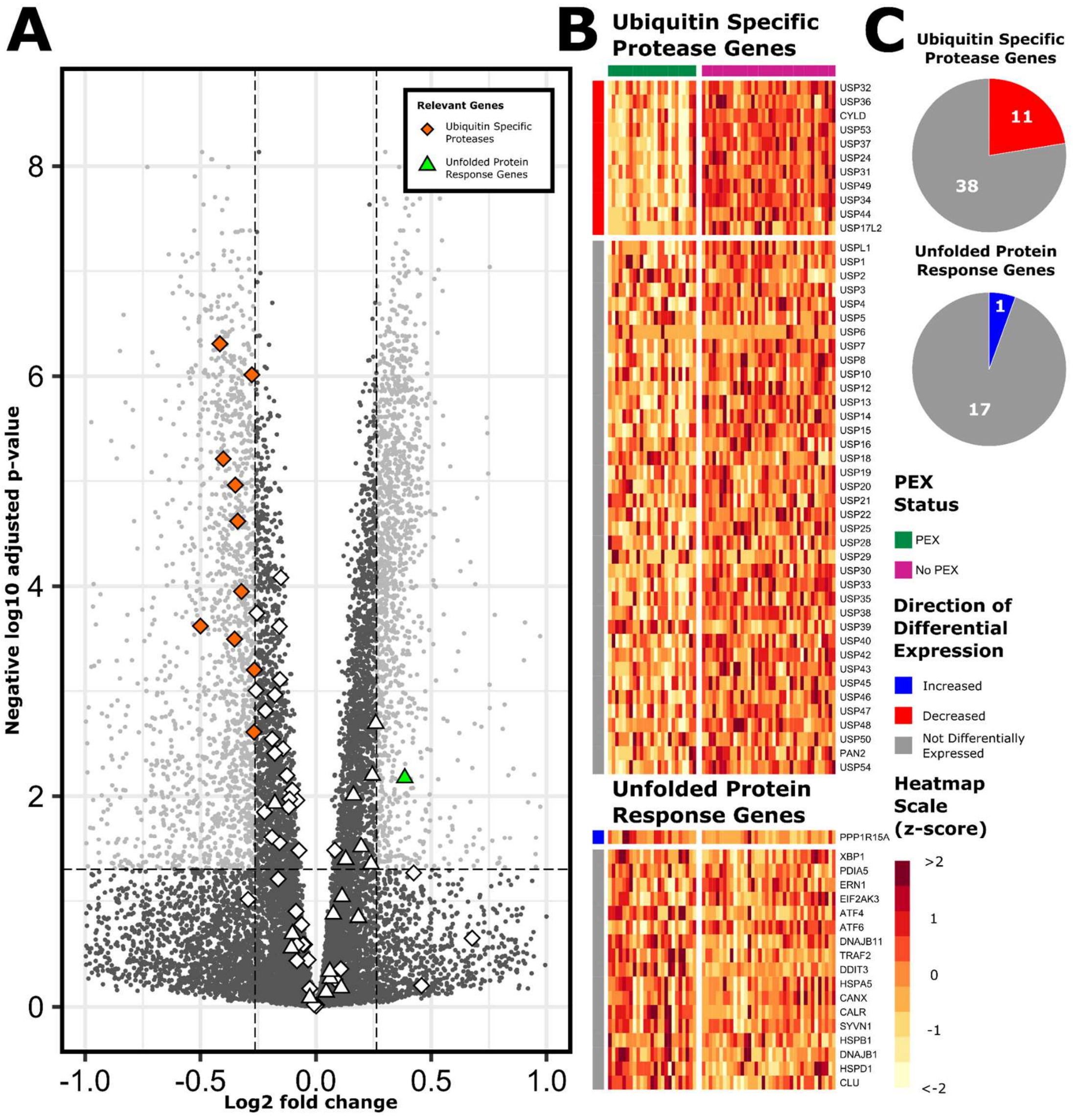

Supplementary Figure S6: Ubiquitin Specific Proteases and Unfolded Protein Response Genes. A volcano plot including all genes encoding ubiquitin specific proteases (USP) and unfolded protein response (UPR) proteins indicates differential expression of all relevant gene transcripts (A). Differentially expressed USP genes are coloured orange and a single differentially expressed UPR gene is coloured light blue. An observed trend toward decreased expression of USP genes is highlighted in a dark grey ellipse. An observed trend toward increased expression of UPR genes is highlighted in a light grey ellipse. Heatmaps (B) demonstrate per sample expression of all genes from these two classes which were measured in this study. Expression is represented as the normalised expression per gene relative to all samples (z-scores). PEX: pseudoexfoliation syndrome; DEG: differentially expressed genes; non DE genes: non-differentially expressed genes; USP: ubiquitin specific proteases; UPR: unfolded protein response.

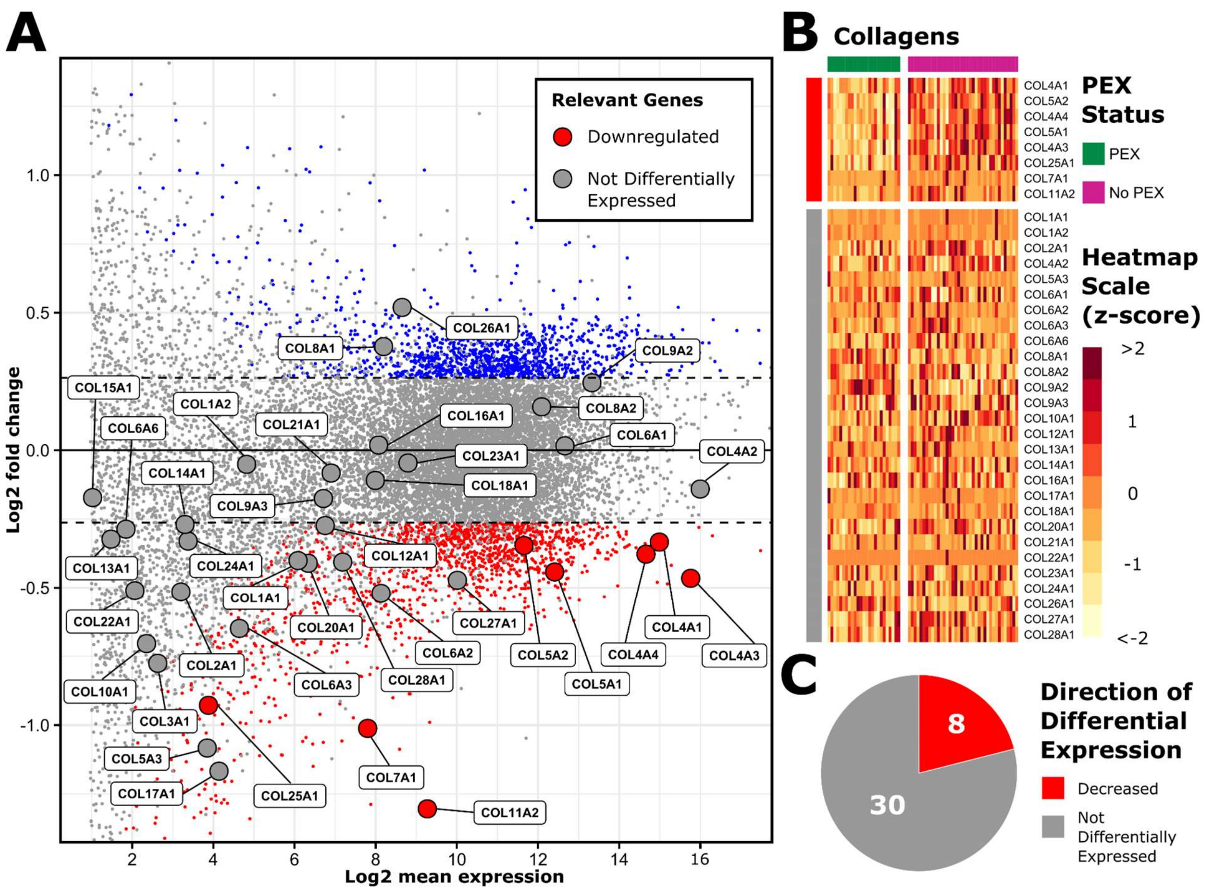

Supplementary Figure S7: Collagen Genes. An MAplot including all collagen genes (A). Horizontal dashed lines represent the log2 fold change cutoff of ± log2(1.2). Differentially expressed genes (defined by a log2 fold change of ± log2(1.2) and padj < 0.05) are represented by blue (upregulated) and red (downregulated) points. Non-differentially expressed genes are represented by grey points. Individual gene expression per sample is represented as the normalised expression (z-score) per gene in class-specific heatmaps (B). PEX: pseudoexfoliation syndrome.

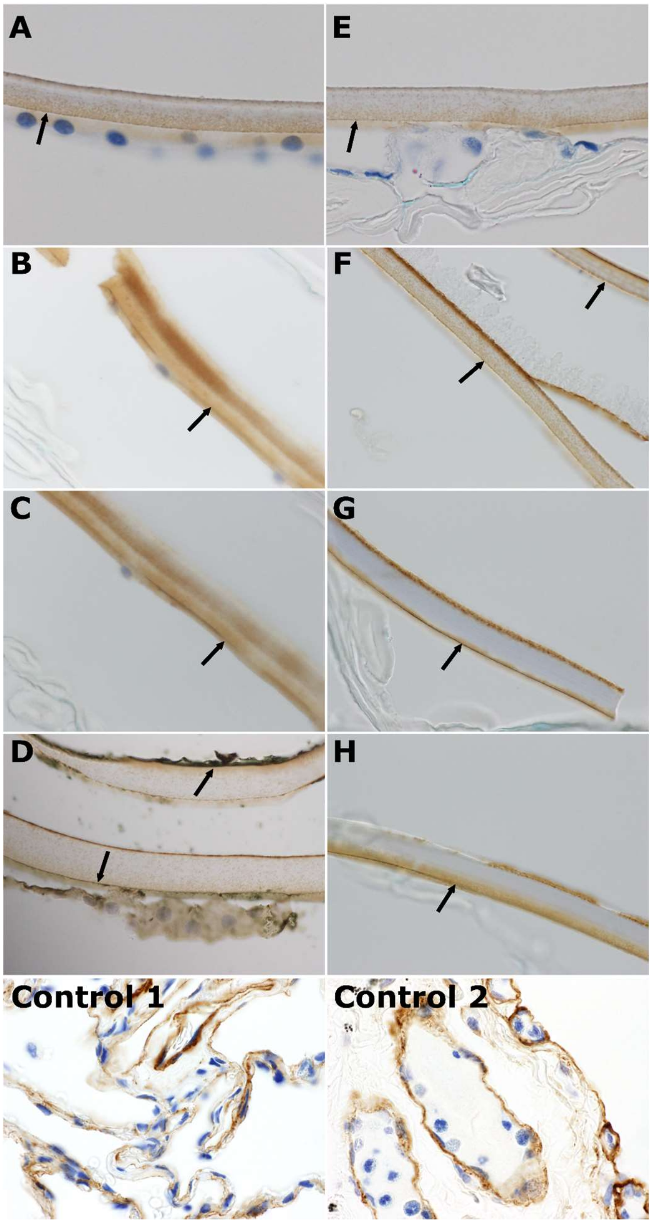

Supplementary Figure S8: Type 4 Collagen Immunohistochemistry. An Immunohistochemical study targeting type 4 collagen demonstrated expected staining in the basal and apical regions of lens capsules which was not discernibly different in intensity between four individuals with PEX (A-D; 60x magnification), and four non-PEX controls (E-H); 60x magnification). Two normal human lung parenchymal specimens were included as positive controls (60x magnification). Arrows indicate the basement membrane at the apical surface of the lens capsule. PEX: pseudoexfoliation syndrome.

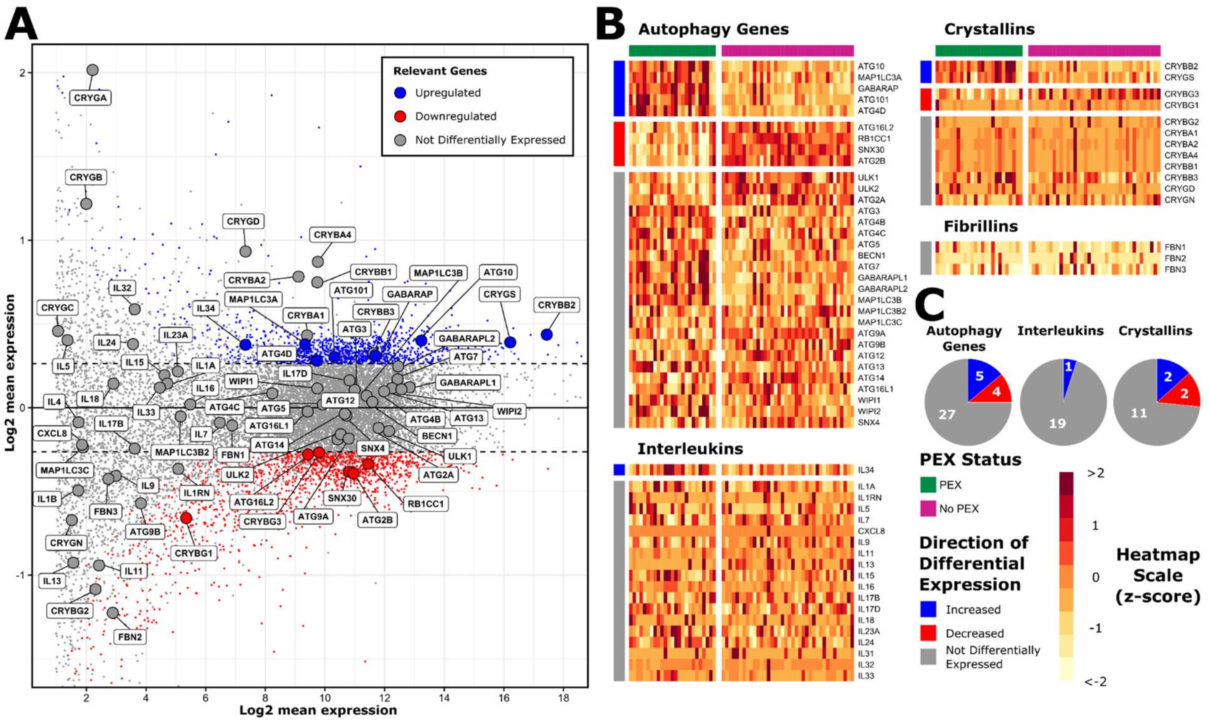

Supplementary Figure S9: Other Relevant Gene Classes. An MAplot including all genes encoding autophagy genes, interleukins, crystallins, and fibrillins demonstrates differential expression of all relevant gene transcripts (A). Differentially expressed genes (defined by a log2 fold change of ± log2(1.2) and padj < 0.05) are represented by blue (upregulated) and red (downregulated) points.Gene expression behaviour is represented by colour: upregulated = blue; downregulated = red; not differentially expressed = grey. Heatmaps (B) demonstrate per sample expression of all genes from these two classes which were measured in this study. Expression is represented as the normalised expression per gene relative to all samples (z-scores). PEX: pseudoexfoliation syndrome.

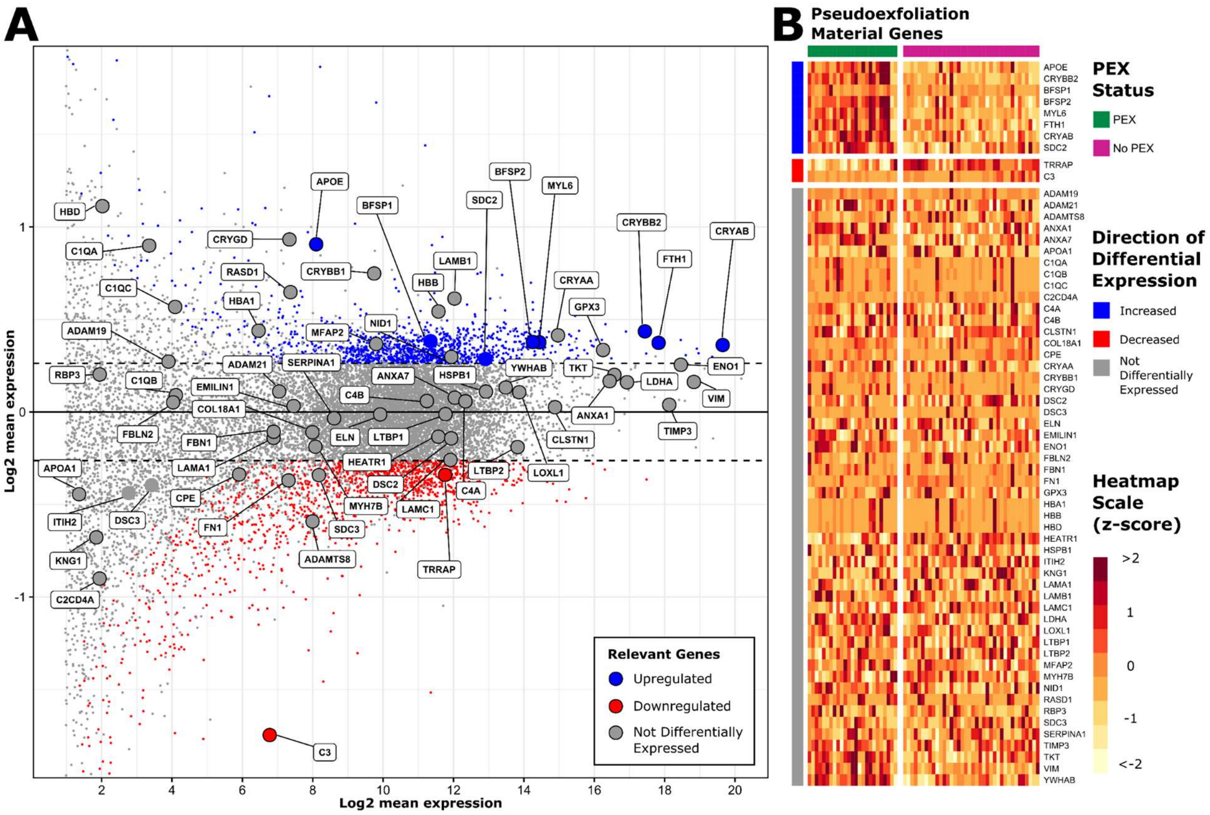

Supplementary Figure S10: Pseudoexfoliation Material Genes. An MAplot including all differentially expressed genes associated with proteins identified in pseudoexfoliation material or aqueous humour (A). Differentially expressed genes (defined by a log2 fold change of ± log2(1.2) and padj < 0.05) are represented by blue (upregulated) and red (downregulated) points. Gene expression behaviour is represented by colour: upregulated = blue; downregulated = red; not differentially expressed = grey. Heatmaps (B) demonstrate per sample expression of all genes from these two classes which were measured in this study. Expression is represented as the normalised expression per gene relative to all samples (z-scores). PEX: pseudoexfoliation syndrome.

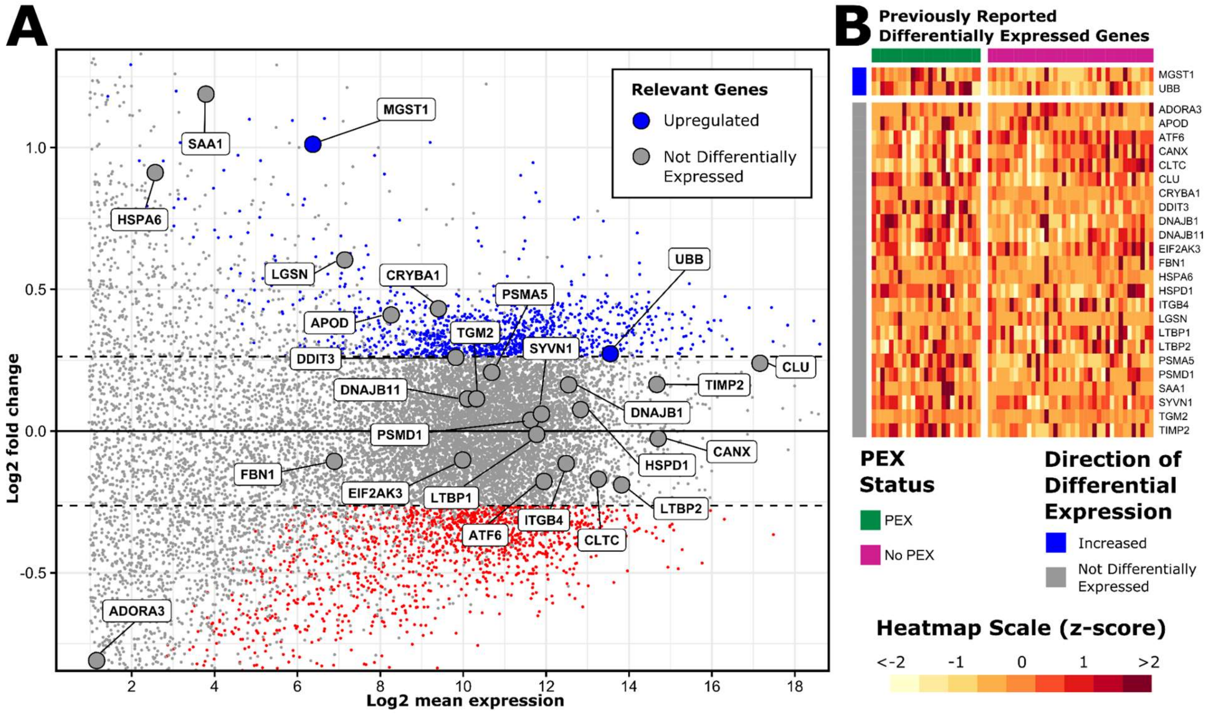

Supplementary Figure S11: Previously identified differentially expressed genes. An MAplot including all genes previously demonstrated to be differentially expressed in PEX ocular biospecimens (A). Horizontal dashed lines represent the log2 fold change cutoff of $\pm$ log2(1.2). Differentially expressed genes (defined by a log2 fold change of $\pm$ log2(1.2) and padj < 0.05) are represented by blue (upregulated) and red (downregulated) points.Gene expression behaviour is represented by colour: upregulated = blue; downregulated = red; not differentially expressed = grey. Heatmaps (B) demonstrate per sample expression of all genes from these two classes which were measured in this study. Expression is represented as the normalised expression per gene relative to all samples (z-scores). PEX: pseudoexfoliation syndrome.

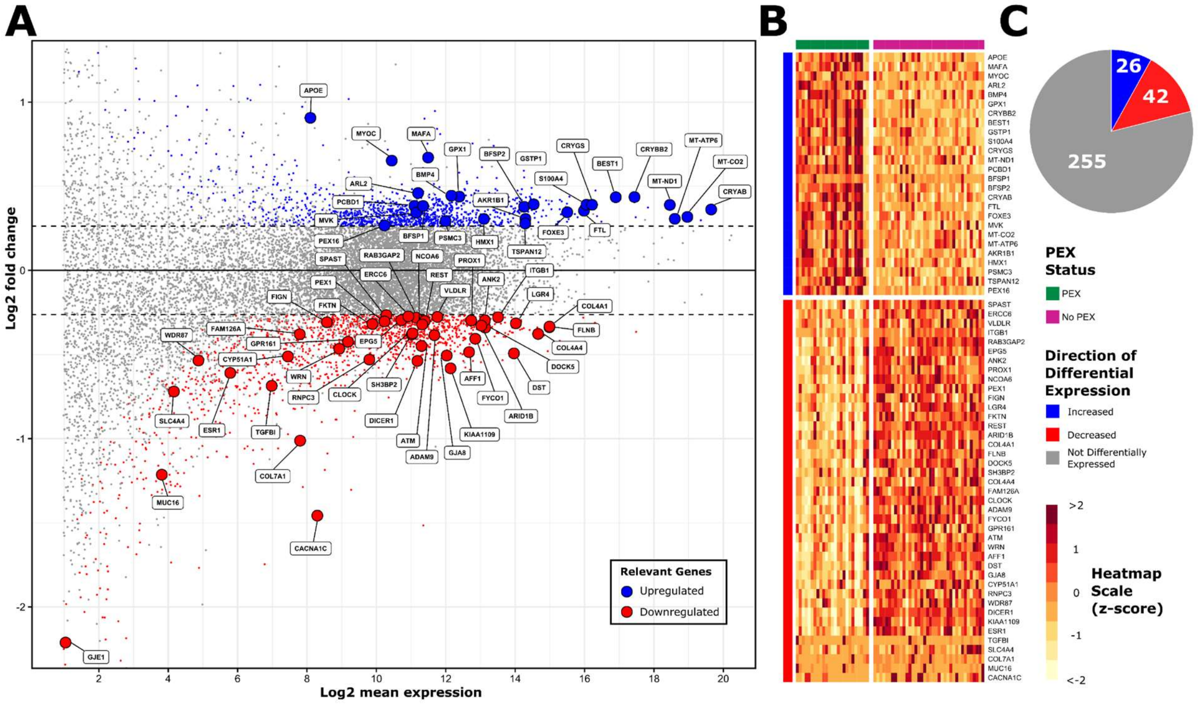

Supplementary Figure S12: Mendelian Cataract Disease-Associated Genes. An MAplot including all differentially expressed Mendelian cataract genes (A). Differentially expressed genes (defined by a log2 fold change of ± log2(1.2) and padj < 0.05) are represented by blue (upregulated) and red (downregulated) points.Gene expression behaviour is represented by colour: upregulated = blue; downregulated = red; not differentially expressed = grey. Heatmaps (B) demonstrate per sample expression of all genes from these two classes which were measured in this study. Expression is represented as the normalised expression per gene relative to all samples (z-scores). PEX: pseudoexfoliation syndrome.

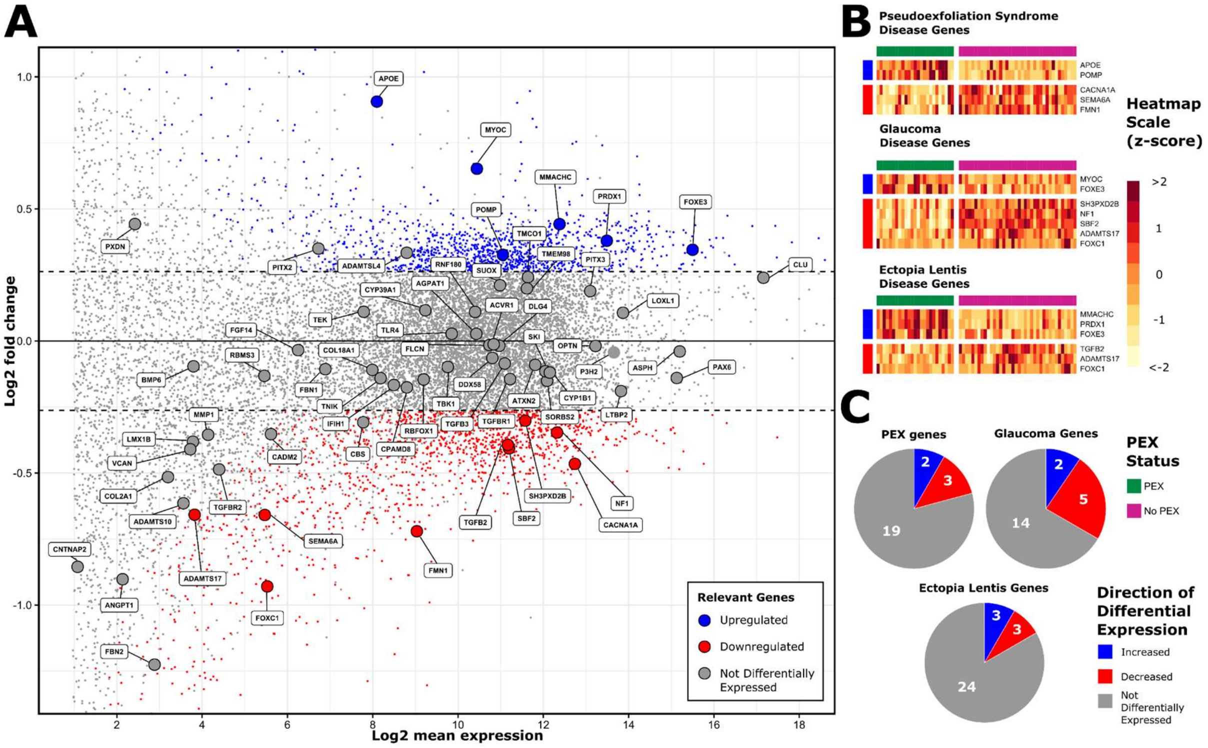

Supplementary Figure S13: Other Mendelian Disease-Associated Genes. An MAplot including all differentially expressed Mendelian cataract genes (A). An MAplot including all collagen genes (A). Horizontal dashed lines represent the log2 fold change cutoff of ± log2(1.2). Differentially expressed genes (defined by a log2 fold change of ± log2(1.2) and padj < 0.05) are represented by blue (upregulated) and red (downregulated) points. Heatmaps (B) demonstrate per sample expression of all genes from these two classes which were measured in this study. Expression is represented as the normalised expression per gene relative to all samples (z-scores). PEX: pseudoexfoliation syndrome.

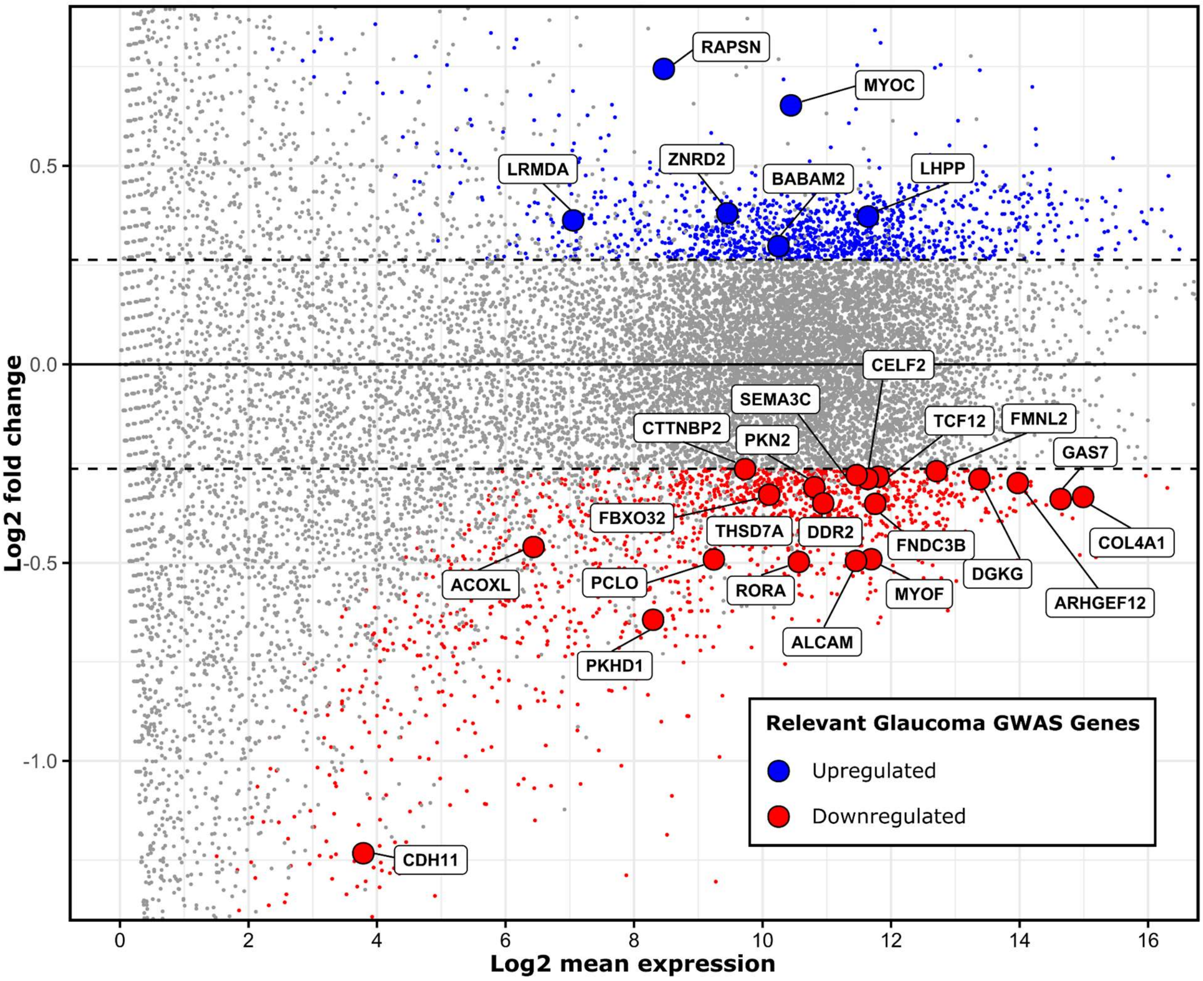

Supplementary Figure S14: Glaucoma GWAS Glaucoma Genes. An MAplot demonstrating differentially expressed glaucoma GWAS genes. The interrogated gene list included all protein coding genes collocated with 127 primary open-angle glaucoma disease-associated single-nucleotide variants identified in a recent cross-ancestry meta-analysis of genome-wide association studies.[1] Horizontal dashed lines represent the log2 fold change cutoff of $\pm$ log2(1.2). Differentially expressed genes (defined by a log2 fold change of $\pm$ log2(1.2) and adjusted p-value < 0.05) are represented by blue (upregulated) and red (downregulated) points. GWAS: genome-wide association study.

APPENDIX - GENE LISTS AND RESOURCES

**Interrogated Gene Classes**
Mitochondrial Respiratory Chain Complex, Mitochondrial Genome, Ribosomal Proteins, G Proteins Coupled Receptors, Heterotrimeric G Proteins, Transforming Growth Factor Beta, Latent Transforming Growth Factors, M10 Matrix Metallopeptidases, Tissue Inhibitors of Matrix Metallopeptidases, Autophagy Genes, Interleukins, Crystallins, Fibrillins, Hallmark Unfolded Protein Response*

Supplementary Table S1: Analysed Gene Classes. Class specific analysis was performed using curated gene lists acquired from the HUGO Gene Nomenclature Committee at the European Bioinformatics Institute in July 2021 (https://www.genenames.org/). *In the absence of a HUGO gene list for unfolded protein response genes, the REACTOME “Hallmark Unfolded Protein Response” gene list was acquired from the Molecular Signatures Database’ (MSigDB v7.2.).

Disease	Source	Genes
Cataract[2]	Cataract Map<br><br>Washington University School of Medicine St Louis	*ABCA3, ABCD1, ABCD3, ABHD12, ACE, ADAM9, ADAMTS10, ADAMTS18, ADAMTSL4, ADIPOQ, ADPCZNC, AFF1, AGK, AGPS, AIC, AIPL1, AIRE, AKR1B1, AKR1E2, ALDH18A1, ALDH1A1, ALG12, ANK2, AP4B1, APOA1, APOE, APP, AQP5, ARCC1, ARID1B, ARL2, ARSE, ASIC2, ATAD3A, ATM, ATOH7, B3GALTL, B3GNT4, B4GALT7, BAP1, BCAR3, BCOR, BEST1, BFSP1, BFSP2, BIN3, BMP4, BRD4, BUB1B, CACNA1C, CAPN15, CASR, CAT, CATC3, CATM, CAV1, CC2D2A, CCA1, CCNP, CCV, CDK5RAP2, CDKN2A, CECR, CHMP4B, CLOCK, CLPB, CNBP, CNGB3, COL11A1, COL18A1, COL2A1, COL4A1, COL4A4, COL4A5, COL4A6, COL7A1, COL9A2, COPB1, CPAMD8, CPOX, CRIM1, CRYAA, CRYAB, CRYBA1, CRYBA2, CRYBA4, CRYBB1, CRYBB2, CRYBB3, CRYGA, CRYGB, CRYGC, CRYGD, CRYGEP1, CRYGFP, CRYGS, CRYZ, CTAA1, CTAA2, CTDP1, CTNND2, CTPL1, CTPP5, CTRCT25, CTRCT29, CTRCT35, CTRCT37, CYP27A1, CYP46A1, CYP51A1, DCR, DHCR7, DICER1, DMPK, DNASE2B, DNMBP, DOCK5, DPAGT1, DST, DYNC1H1, EBP, EFNA5, EPG5, EPHA2, ERCC2, ERCC6, ERCC8, ESCO2, ESR1, ETFDH, EYA1, EZR, FAM126A, FBN1, FDFT1, FIGN, FKRP, FKTN, FLNB, FOXE3, FTL, FTO, FYCO1, FZD4, GALE, GALK1, GALT, GBA2, GCM2, GCNT2, GEMIN4, GFER, GJA1, GJA3, GJA8, GJB6, GJE1, GLA, GNAS, GNPAT, GPR161, GPX1, GSTM1, GSTP1, GSTT1, GTF2IRD1, GUCY2D, HCCS, HIP1, HMX1, HRAS, HSF4, HSPG2, IARS2, ICA1, IDO1, IFNGR1, IGF1R, IKBKG, INPP5K, INS, INTS1, IPO13, ITGB1, ITM2B, ITPA, JAM3, JBS, KCNJ13, KIAA1109, KLC1, KNO3, KONDS, LAMB2, LAMP2, LARGE, LCA5, LCT, LCTL, LEMD2, LGR4, LGSN, LIM2, LMX1B, LONP1, LOXL1, LRP2, LRP5, LRP5L, LSS, LTBP2, MAB21L2, MAF, MAFA, MAN2B1, MIP, MIR184, MMP1, MMP2, MT-ATP6, MT-CO2, MT-CYB, MTHFR, MT-ND1, MT-ND4, MT-ND5, MT-TI, MT-TL1, MT-TP, MT-TW, MUC16, MVK, MYH9, MYO7A, MYOC, MYP4, NAT2, NBN, NCOA6, NDP, NEIL2, NEK9, NEU1, NF2, NHS, NID1, NOD2, NOG, NPHP1, NR2E3, NRCAM, NSUN2, NUP188, OAT, OCRL, OGG1, OPA1, OPA3, OSGEP, OTX2, P2RY2, P3H2, PANK4, PARP1, PAX6, PCBD1, PDE6B, PEX1, PEX10, PEX11B, PEX12, PEX13, PEX14, PEX16, PEX26, PEX3, PEX5, PEX6, PEX7, PITX2, PITX3, PNPT1, POLG, POLR3B, POMGNT1, POMT1, POMT2, PON2, PORCN, PQBP1, PROX1, PRX, PSMC3, PTCH1, PTEN, PTH, PTN, PVRL3, PXDN, PXMP3, PYCR1, RAB3GAP1, RAB3GAP2, RECQL4, REST, RGS6, RIC1, RIN2, RNF149, RNLS, RNPC3, RP2, RPE65, RPGR, RRAGA, S100A4, SALL4, SC5D, SCFD2, SCHIP1, SEC23A, SH3BP2, SIL1, SIPA1L3, SIX5, SIX6, SLC11A2, SLC16A12, SLC23A1, SLC25A15, SLC33A1, SLC40A1, SLC4A4, SLC7A8, SMAD4, SMARCA4, SMO, SORD, SOX11, SOX2, SPARC, SPAST, SRD5A3, SSTR2, TAF1A, TAPT1, TBC1D20, TDRD7, TELO2, TENM3, TFAP2A, TGFB3, TGFBI, TLR3, TMCO3, TMEM114, TOR1AIP1, TP53, TRAPPC11, TRNT1, TRPM3, TSPAN12, TSR1, TUBA1A, UBE2A, ULK4, UNC45B, USH1C, USH2A, VCAN, VIM, VLDLR, VSX2, WBS2, WDR36, WDR87, WFS1, WNT3, WRN, XPC, XRCC1, XYLT2, YAP1, YWHAE, ZNF350, ZNF526*
PEX[3–13]*	GWAS / EWAS	*LOXL1, POMP, TMEM136, AGPAT1, RBMS3, SEMA6A, CACNA1A, OR11L1, CD80, TNIK, CADM2, SORBS2, RNF180, FGF14, FMN1, RBFOX1, CLU, CNTNAP2, APOE, GST, TNFA, MMP1, TLR4, LTBP2, CTGF, TMCO1, CDKN2BAS, ATXN2, TBC1D21, PON1, IL10, BMP6, ACVR1, FBLN5, CYP39A1*
Glaucoma**	Genomics England	*ADAMTS10, ADAMTS17, ANGPT1, CPAMD8, CYP1B1, DDX58, FOXC1, FOXE3, IFIH1, LMX1B, LTBP2, MYOC, NF1, OCRL, OPTN, PAX6, PITX2, SBF2, SH3PXD2B, TBK1, TEK, TMEM98*
Glaucoma GWAS***	Gharahkhani et al. (2021)	*RERE, TRAPPC3, RSPO1, GLIS1, CDC7, MOV10, DDR2, TMCO1, MYOC, TRIB2, BABAM2, PRKCE, PNPT1, SPRED2, ANTXR1, ZNF638, ACOXL, FMNL2, THRB, RARB, ARHGEF3, CADM2, ALCAM, MECOM, FNDC3B, DGKG, LPP, AFAP1, SCFD2, FAM13A, STOX2, ANKH, HLA-G, SRSF3, CLIC5, TFAP2B, GJA1, SLC2A12, PDE7B, TMEM181, THSD7A, CREB5, POU6F2, SEMA3C, PCLO, RELN, CAV1, CTTNBP2, CALD1, PRKAG2, ANGPT2, GTF2E2, ANGPT1, FBXO32, CDKN2B, ABCA1, SVEP1, LMX1B, ABO, CELF2, BICC1, LRMDA, CYP26A1, PLCE1, LHPP, PLEKHA7, RAPSN, ZNRD2, ME3, YAP1, CADM1, TLCD5, ETS1, ADAMTS8, PTHLH, TMTC2, RIC8B, ATXN2, KLF5, LMO7, LTBP2, TTLL5, SYNE3, TCF12, SMAD6, LOXL1, SLCO3A1, SALL1, CDH11, ADAMTS18, SMG6, GAS7, MAPT, NPEPPS,*

		BCAS3, EYA2, GABPA, TXNRD2, CHEK2, TRIOBP, PKN2, TGFBR3, EFEMP1, TSC22D2, PITX2, PKHD1, HSF2, SEMA3E, LSM8, MCPH1, MYOF, PLEKHS1, ARHGEF12, CCDC91, SLC6A15, COL4A1, AREL1, RORA, SV2B, NUDT7, APP, PSMG1
Ectopia Lentis	OMIM	*ADAMTS10, ADAMTS17, ADAMTSL4, ASPH, CBS, COL18A1, COL2A1, CPAMD8, CYP1B1, DLG4, FBN1, FBN2, FLCN, FOXC1, FOXE3, LTBP2, MMACHC, P3H2, PAX6, PITX2, PITX3, PORCN, PRDX1, PXDN, SKI, SUOX, TGFB2, TGFB3, TGFBR1, TGFBR2, VCAN*

Supplementary Table S2: Disease-Associated Genes. Pseudoexfoliation syndrome disease associations were analysed by investigating differential expression of genes implicated in primary genetic causes of these phenotypes. Cat-Map genes were acquired from https://cat-map.wustl.edu/. *Pseudoexfoliation syndrome genes were defined by the genes colocated with disease associated single nucleotide variants implicated in genome-wide association studies, with the addition of *CYP39A1* which was implicated in an exome-wide rare variant association study. **Glaucoma genes were collated from the Genomics England panel (https://panelapp.genomicsengland.co.uk/panels/249/), and comprised all genes reported as having high evidence of association. Additional genes added to this list by the authors of the current study included *ANGPT1*, *OPTN*, and *TBK1*. ***Glaucoma GWAS genes include all protein coding genes collocated with Primary Open-Angle Glaucoma disease variants identified in a large cross-ancestry meta-analysis of genome wide-association studies.[1]PEX: pseudoexfoliation syndrome; GWAS: genome wide association study; EWAS: exome wide association study; OMIM: The 'Online Mendelian Inheritance in Man' database.

Relevant Reference	Protein Product associated Gene
Streeten et al. (1986)[14]	*EMILIN1*
Li et al. (1988)[15]	*APCS, ELN*
Konstas et al. (1990)[16]	*LAMA1, LAMB1, LAMC1*
Schlotzezer-Schrehardt et al. (1992)[17]	*APCS, FN1, LAMA1, LAMB1, LAMC1, NID1, SDC2*
Vogiatzis et al. (1994)[18]	*EMILIN1*
Schlotzezer-Schrehardt et al. (1997)[19]	*FBN1*
Schlotzezer-Schrehardt et al. (2001)[20]	*LTBP2*
Zenkel et al. (2005)[21]	*FBN1, LTBP1, LTBP2*
Schlotzezer-Schrehardt et al. (2006)[22]	*MFAP2, VTN*
Ovodenko et al. (2007)[23]	*ADAM19, ADAM21, ADAMTS8 ,APCS, C1QA, C1QB, C1QC, C3, C4A, C4B, CLU, DSC2, DSC3, FBLN2, FBN1, FN1, LAMA1, LAMB1, LAMC1, NID1, SDC3, TIMP3, VCN, VTN*
Schlotzezer-Schrehardt et al. (2008)[24]	*LOXL1*
Sharma et al. (2009)[25]	*LTBP2, C3, CLU , APOE, LOXL1*
Sharma et al. (2018)[26]	*ALDH3A1, ANXA1, ANXA7, APOA1, APOA2, APOA4, APOC3, APOE, ARHGAP42, BFSP1, BFSP2, C2CD4A, C3, CLU, COL18A1, CRYAA, CRYAB, CRYBB2, EMILIN1, ENO1, FBN1, FGA, FGB, FGG, FN1, FTH1, HBA1, HBB, HBD, HBE1, HBG1, HEATR1, HIST1H2BO, HIST1H4A, HIST2H2AC, HSPB1, IGHG1, IGKC, ITIH2, ITIH4, KERA, LDHA, LOXL1, LTBP2, MRVI1, MYH7B, MYH9, MYL6, PKM, PRDX2, PTPN5, RASD1, S100A6, SASH1, SERPINA1, SLC4A1, SPTA1, SPTB, TIMP3, TKT, TRIM31, TRRAP, VIM, VTN, YWHAB*
Botling Taube et al. (2019)*[27]	**[Upregulated]** *C3, AMBP, KNG1, SERPINC1, GC, CRYGD, CRYBB1, CRYBB2*<br>**[Downregulated]** *CLSTN1, RBP2, CPE, GPX3*

Supplementary Table S3: Genes Associated with Proteins Identified in Pseudoexfoliation Material. The data within this table was collated from a literature search performed to identify studies in which pseudoexfoliation material proteins were identified using protein or proteomic analysis. *Genes listed from a mass spectrometry study by Botling Taube et al. (2019) correspond to proteins identified in the aqueous humour of patients with PEX.

Relevant Reference	Differentially Expressed Transcripts
Schlotzezer-Schrehardt et al. (1997)[19]	*FBN1*
Schlotzezer-Schrehardt et al. (2001)[20]	*LTBP2*
Zenkel et al. (2005)[21]	*ADORA3, AKAP2, APOD, CLTC, CRYBA1, FBN1, LGSN, LTBP1, LTBP2, MGST1, SAA1, TGM2, TIMP1, TIMP2*
Ovodenko et al. (2007)[23]	*CLU, FBN1*
Schlotzezer-Schrehardt et al. (2008)[24]	*LOXL1*
Sharma et al. (2009)[25]	*CLU*
Khan et al. (2010)[28]	*LOXL1*
Hayat et al. (2019)[29]	*EIF2AK3, ATF6, DNAJB11, DDIT3, CASP12, HSPA6, CANX, SYVN1, DNAJB1, HSPD1, UBB, PSMD1, PSMA5*

Supplementary Table S4: Previously Identified Different Expressed Genes in Pseudoexfoliation Syndrome. The data within this table was collated from a literature search performed to identify studies in which mRNA expression was measured in ocular biospecimens collected from individuals affected by pseudoexfoliation syndrome.

GENE RESOURCE REFERENCES

1. Gharahkhani P, Jorgenson E, Hysi P, et al. Genome-wide meta-analysis identifies 127 open-angle glaucoma loci with consistent effect across ancestries. *Nat Commun*. 2021;12(1):1258.

2. Shiels A, Bennett TM, Hejtmancik JF. Cat-Map: putting cataract on the map. *Mol Vis*. 2010;16:2007-2015.

3. Thorleifsson G, Magnusson KP, Sulem P, et al. Common sequence variants in the LOXL1 gene confer susceptibility to exfoliation glaucoma. *Science*. 2007;317(5843):1397-1400.

4. Aung T, Ozaki M, Mizoguchi T, et al. Corrigendum: a common variant mapping to CACNA1A is associated with susceptibility to exfoliation syndrome. *Nat Genet*. 2015;47(6):689.

5. Aung T, Ozaki M, Lee MC, et al. Genetic association study of exfoliation syndrome identifies a protective rare variant at LOXL1 and five new susceptibility loci. *Nat Genet*. 2017;49(7):993-1004.

6. Zagajewska K, Piątkowska M, Goryca K, et al. GWAS links variants in neuronal development and actin remodeling related loci with pseudoexfoliation syndrome without glaucoma. *Exp Eye Res*. 2018;168:138-148.

7. Aboobakar IF, Allingham RR. Genetics of exfoliation syndrome and glaucoma. *Int Ophthalmol Clin*. 2014;54(4):43-56.

8. Aboobakar IF, Johnson WM, Stamer WD, Hauser MA, Allingham RR. Major review: Exfoliation syndrome; advances in disease genetics, molecular biology, and epidemiology. *Exp Eye Res*. 2017;154:88-103.

9. Ma YN, Xie TY, Chen XY. Multiple Gene Polymorphisms Associated with Exfoliation Syndrome in the Uygur Population. *J Ophthalmol*. 2019;2019:9687823.

10. Can Demirdöğen B, Koçan Akçin C, Göksoy E, et al. Paraoxonase 1 (PON1) promoter (-107T/C) and coding region (192Q/R and 55L/M) genetic variations in pseudoexfoliation syndrome and pseudoexfoliative glaucoma risk. *Graefes Arch Clin Exp Ophthalmol*. 2019;257(10):2257-2270.

11. Fakhraie G, Parvini F, Ghanavi J, Saif S, Farnia P. Association of IL-10 gene promoter polymorphisms with susceptibility to pseudoexfoliation syndrome, pseudoexfoliative and primary open-angle glaucoma. *BMC Med Genet*. 2020;21(1):32.

12. Kondkar AA, Sultan T, Azad TA, Osman EA, Almobarak FA, Al-Obeidan SA. Association analysis of polymorphisms rs12997 in ACVR1 and rs1043784 in BMP6 genes involved in bone morphogenic protein signaling pathway in primary angle-closure and pseudoexfoliation glaucoma patients of Saudi origin. *BMC Med Genet*. 2020;21(1):145.

13. Genetics of Exfoliation Syndrome Partnership, Li Z, Wang Z, et al. Association of Rare CYP39A1 Variants With Exfoliation Syndrome Involving the Anterior Chamber of the Eye. *JAMA*. 2021;325(8):753-764.

14. Streeten BW, Gibson SA, Dark AJ. Pseudoexfoliative material contains an elastic microfibrillar-associated glycoprotein. *Trans Am Ophthalmol Soc*. 1986;84:304-320.

15. Li ZY, Streeten BW, Wallace RN. Association of elastin with pseudoexfoliative material: an immunoelectron microscopic study. *Curr Eye Res*. 1988;7(12):1163-1172.

16. Konstas AG, Marshall GE, Lee WR. Immunogold localisation of laminin in normal and exfoliative iris. *Br J Ophthalmol*. 1990;74(8):450-457.

17. Schlötzer-Schrehardt U, Dörfler S, Naumann GO. Immunohistochemical localization of basement membrane components in pseudoexfoliation material of the lens capsule. *Curr Eye Res*. 1992;11(4):343-355.

18. Vogiatzis A, Marshall GE, Konstas AG, Lee WR. Immunogold study of non-collagenous matrix components in normal and exfoliative iris. *Br J Ophthalmol*. 1994;78(11):850-858.

19. Schlötzer-Schrehardt U, von der Mark K, Sakai LY, Naumann GO. Increased extracellular deposition of fibrillin-containing fibrils in pseudoexfoliation syndrome. *Invest Ophthalmol Vis Sci*. 1997;38(5):970-984.

20. Schlötzer-Schrehardt U, Zenkel M, Küchle M, Sakai LY, Naumann GO. Role of transforming growth factor-beta1 and its latent form binding protein in pseudoexfoliation syndrome. *Exp Eye Res*. 2001;73(6):765-780.

21. Zenkel M, Pöschl E, von der Mark K, et al. Differential gene expression in pseudoexfoliation syndrome. *Invest Ophthalmol Vis Sci*. 2005;46(10):3742-3752.

22. Schlötzer-Schrehardt U, Naumann GOH. Ocular and Systemic Pseudoexfoliation Syndrome. *American Journal of Ophthalmology*. 2006;141(5):921-937.e2. doi:10.1016/j.ajo.2006.01.047

23. Ovodenko B, Rostagno A, Neubert TA, et al. Proteomic analysis of exfoliation deposits. *Invest Ophthalmol Vis Sci*. 2007;48(4):1447-1457.

24. Schlötzer-Schrehardt U, Pasutto F, Sommer P, et al. Genotype-correlated expression of lysyl oxidase-like 1 in ocular tissues of patients with pseudoexfoliation syndrome/glaucoma and normal patients. *Am J Pathol*. 2008;173(6):1724-1735.

25. Sharma S, Chataway T, Burdon KP, et al. Identification of LOXL1 protein and Apolipoprotein E as components of surgically isolated pseudoexfoliation material by direct mass spectrometry. *Experimental Eye Research*. 2009;89(4):479-485. doi:10.1016/j.exer.2009.05.001

26. Sharma S, Chataway T, Klebe S, et al. Novel protein constituents of pathological ocular pseudoexfoliation syndrome deposits identified with mass spectrometry. *Mol Vis*. 2018;24:801-817.

27. Botling Taube A, Konzer A, Alm A, Bergquist J. Proteomic analysis of the aqueous humour in eyes with pseudoexfoliation syndrome. *Br J Ophthalmol*. 2019;103(8):1190-1194.

28. Khan TT, Li G, Navarro ID, et al. LOXL1 expression in lens capsule tissue specimens from individuals with pseudoexfoliation syndrome and glaucoma. *Mol Vis*. 2010;16:2236-2241.

29. Hayat B, Padhy B, Mohanty PP, Alone DP. Altered unfolded protein response and proteasome impairment in pseudoexfoliation pathogenesis. *Exp Eye Res*. 2019;181:197-207.
